# Extracellular vesicles of *Trypanosoma cruzi* and immune complexes they form with sialylated and non-sialylated IgGs increase small peritoneal macrophage subpopulation and elicit different cytokines profiles

**DOI:** 10.3389/fimmu.2023.1215913

**Published:** 2023-08-02

**Authors:** Alberto Cornet-Gomez, Lissette Retana Moreira, Mercedes Gomez-Samblás, Antonio Osuna

**Affiliations:** ^1^ Grupo de Bioquímica y Parasitología Molecular (CTS 183), Departamento de Parasitología, Instituto de Biotecnología, Universidad de Granada, Granada, Spain; ^2^ Departamento de Parasitología, Facultad de Microbiología, Universidad de Costa Rica, San José, Costa Rica; ^3^ Centro de Investigación en Enfermedades Tropicales (CIET), Universidad de Costa Rica, San José, Costa Rica

**Keywords:** extracellular vesicles, trypomastigotes, *Trypanosoma cruzi*, macrophages, interleukins, immune modulation

## Abstract

American trypanosomiasis, or Chagas disease, is caused by the protozoan parasite *Trypanosoma cruzi* and is characterized by the presence of cardiac or gastrointestinal symptoms in a large number of patients during the chronic phase of the disease. Although the origin of the symptoms is not clear, several mechanisms have been described involving factors related to *T. cruzi* and the host immune response. In this sense, the extracellular vesicles (EVs) secreted by the parasite and the immune complexes (ICs) formed after their recognition by host IgGs (EVs-IgGs) may play an important role in the immune response during infection. The aim of the present work is to elucidate the modulation of the immune response exerted by EVs and the ICs they form by analyzing the variation in the subpopulations of small and large peritoneal macrophages after intraperitoneal inoculation in mice and to evaluate the role of the sialylation of the host IgGs in this immunomodulation. Both macrophage subpopulations were purified and subjected to cytokine expression analysis by RT-qPCR. The results showed an increase in the small peritoneal macrophage subpopulation after intraperitoneal injection of parasite EVs, but a greater increase in this subpopulation was observed when sialylated and non-sialylated ICs were injected, which was similar to inoculation with the trypomastigote stage of the parasite. The cytokine expression results showed the ability of both subpopulations to express inflammatory and non-inflammatory cytokines. These results suggest the role of free EVs in the acute phase of the disease and the possible role of immune complexes in the immune response in the chronic phase of the disease, when the levels of antibodies against the parasite allow the formation of immune complexes. The differential expression of interleukins showed after the inoculation of immune complexes formed with sialylated and non-sialylated IgGs and the interleukins expression induced by EVs, demonstrates that the IgG glycosilation is involved in the type of immune response that dominates in each of the phases of the Chagas disease.

## 1 Introduction

*Trypanosoma cruzi* (*T. cruzi*) is the causative agent of American trypanosomiasis or Chagas disease, considered a neglected infectious disease by the World Health Organization. Recent studies estimate that there are 6-7 million people infected with this parasite, approximately 14,000 deaths per year by causes related to the infection, and approximately 80 million people at risk of infection (1, 2). Chagas disease was initially restricted to geographical areas where the natural biological cycle of the parasite was maintained, comprising 21 countries in the Americas; however, it has become a global health issue as a result of migratory movements. Besides transmission through contact with feces of infected triatomine bugs of the family Reduviidae (subfamily Triatominae), infection with the parasite can occur by oral, transfusional or through vertical routes, epidemiologically important in areas where vector-borne transmission does not occur (3, 4).

The course of Chagas disease presents two phases: acute and chronic. The acute phase corresponds to the first weeks of infection with this intracellular parasite, in which no clear pathognomonic symptoms are present, but high parasitemia can be observed. The chronic phase appears from the 3^rd^ to the 8^th^ week after infection and, during this phase, the presence of trypomastigotes (the infective stage of the parasite for the vertebrate host) in blood is drastically reduced; however, the individual remains infected for decades. It is estimated that only 30-40% of infected individuals will develop characteristic symptoms of the disease during the chronic phase (determinate form of the disease), including cardiac involvement or gastrointestinal megasyndromes (5, 6).

During an infection with *T. cruzi*, the immune system plays a crucial role; in this sense, the innate immune response is able to detect the pathogen-associated molecular patterns (PAMPs) present on the parasite`s surface, most of which are the glycosylphosphatidylinositol anchors derived from *T. cruzi* mucin-like glycoproteins (GPI-mucins). These molecules are recognized by Toll-like receptors (TLRs), which are part of a group of receptors called pattern recognition receptors (PRRs) (7, 8). Stimulation of TLRs is important in controlling parasitemia through the production of cytokines such as IL-12 and IFN-γ. During the acute phase of the disease, an increased production of inflammatory cytokines (IL-12, IL-6, IFN-γ and TNF-α) and chemokines (CCL2, CCL3, CCL4, CCL5 and CXCL10) is observed. Following PAMP recognition, antigen presenting cells (APCs) initiate the adaptive immune response necessary to control parasitemia, leading to the production of specific antibodies and the activation of CD4+ and CD8+ T lymphocytes. Moreover, during this phase, the inflammatory response (Th1 and Th17) also leads to the production of nitric oxide (NO), and this response is related to the resistance to *T. cruzi* infection and control of parasitemia (9–11). Although the inflammation process elicited during the acute phase of the disease is able to control parasitemia, it does not completely eliminate the parasite, leading to the chronic phase, in which a fine balance between inflammatory and anti-inflammatory cytokines and an effective cellular response needs to be reached to keep parasite levels in check while avoiding tissue damage (12).

Macrophages are considered to be part of the first barrier to prevent infections, acting as antigen-presenting cells to CD4+ T lymphocytes. Under normal physiological conditions, monocytes present a quiescent state (M0: CCL1-, CD163-, CD14+) (13); however, macrophages have been classified as M1 and M2, the former based on classical activation, and M2 designated for alternatively activated macrophages. M1 macrophages are mostly induced by TLR ligands (bacterial lipopolysaccharides, LPS) or by some cytokines, such as IFN-γ, TNF-α and GM-CSF. On the other hand, M2 macrophages refer to alternatively activated macrophages and can be polarized by various stimulatory factors, such as cytokines (IL-4, IL-10 and IL-13), glucocorticoids or immune complexes and LPS. This macrophage classification into M1 and M2 is maintained despite evidence that M2 designation encompasses cells with different physiological and biochemical patterns (14). Moreover, in 2010, Ghosn et al. described two subpopulations of peritoneal macrophages with physically, functionally and evolutionarily different immunological markers: i.) large peritoneal macrophages (LPM), of embrionary origin and that express high levels of CD11b and F4/80, and ii.) small peritoneal macrophages (SPM) of monocytic origin and that express lower levels of CD11b and F4/80 but higher levels of MHC-II. LPM are the majority subpopulation in unstimulated animals but, upon inflammatory stimuli, for example, with LPS, thyoglicolate or after inoculation of *T. cruzi* flagellate forms, SPM become the majority subpopulation (15, 16). SPM, but not LPM, have the ability to present antigens to naive CD4+ T cells *via* the activating receptor DNAM-1 (CD226) (17). These authors propose that SPM are functionally distinct from LPM and that DNAM-1 plays a co-stimulatory role in antigen presentation by SPM. An increased phagocytic capacity of SPM and differences in cytokine production have also been reported. Despite these facts, the role of each macrophage subset has never been described, making it impossible to fit them into the classical M1/M2 classification (18).

IgGs are traditionally recognized as mediators of the humoral immune response. Antibodies bind and neutralize antigens by their Fab region to promote complement-dependent cytotoxicity resulting from binding of antibodies *via* the Fab region to antigen epitopes and binding of Fc regions to complement, binding to specific receptors (FcR) present on the cell membranes of lymphoid cells and in particular of macrophages, which allows the opsonization of antigens and the initiation of phagocytosis of immune complexes by these phagocytic cells.

Based on these receptors present on the surface of macrophages, the effector functions initiated by antibodies and recognized as proinflammatory mediators of the humoral immune response are triggered by the Fc domain upon binding to FcR receptors. Fc γ receptor IIb (FcγRIIb) is the only inhibitory FcγR in the FcγR family (19). These effector functions are largely dependent on the N-terminally bound biantennary glycan of IgG heavy chains, which is located below the Ig hinge region (20, 21). This glycan is considered to hold the two Fc heavy chains in an open conformation necessary to interact with activating Fcγ receptors (FcγRs). However, the presence in the sugar chain of terminal sialic acid in the glycan has profound implications on the effector functions of Fc by inhibiting activation by cells (22), and it is the FcRγIIb receptors that would be responsible for binding to such immunoglobulins carrying sialic acid in their Fc region (23). The presence of sialic acid, bound in an α2,6 bond to the penultimate terminal galactose of the glycan reduces FcγR binding and converts IgG antibodies to anti-inflammatory mediators demonstrated activity (24, 25). Thus, glycosylated IgGs in Fc would be responsible for the *in vivo* anti-inflammatory activity of Intravenous immunoglobulin (IVIg) therapy (24). Where immune complexes with sialylated Fcs initiate an anti-inflammatory cascade *via* the receptor or lectin SIGN-R1 (26). Instead of binding to the FcγR, sialylated Fcs may rather interact with inflammatory cells by the FcR inhibitor, FcγRIIb, or *via* the lectin receptor SIGN-R1 or DC-SIGN and the initiate an anti-inflammatory cascade. This leads to an inhibitory FcγRIIb surface expression on inflammatory cells, thus attenuating the inflammation initiated by autoantibodies in autoimmune diseases where this type of therapy has been used (21). In addition, other authors have recently proposed the hypothesis that non-fucosylated IgGs in serum can saturate FcgRIIIa in immune cells due to its high affinity for the receptor and thus modulate immune responses, demonstrating how the anti-inflammatory activity of IVIG is mediated through activating blockade of the FcgR by galactosylated IgGs, modulating by inhibiting antibody dependent cellular cytotoxicity (ADCC). and complement-dependent cytotoxicity (27).

Extracellular vesicles (EVs) are small membrane-coated vesicles released into the extracellular milieu by all cell types, and classified according to their size, biogenesis and composition (28). In *T. cruzi*, the cargo of EVs is complex and includes nucleic acids (DNA, RNA and miRNA), proteins, lipids and different metabolites (29–32). In previous studies, our research group characterized some of the biological properties of EVs released by different stages of the parasite, studying some of their physical properties, the effects of the interaction between these EVs and cells at the molecular level, the localization and presence of *trans-*sialidases, the formation of immune complexes EVs-IgGs in the plasma of patients and the role they play in inhibiting the complement system; the presence of immune complexes in patients sera means that they could play a role a systemic level (33–36). Garcia-Silva et al. (37) studied the changes induced by EVs secreted by non-infective stages of *T. cruzi* over HeLa cells, finding that the expression of inflammatory interleukins IL-1, IL-6 and IL-18 was increased even after the stimulation with EVs secreted by these non-infective forms. Other groups have also evidenced the ability of EVs of the parasite to induce the release of proinflammatory cytokines (TNF-α and IL-6) and NO production by murine macrophages by interacting with TLR2 (38, 39). In this sense, it is well known that *T. cruzi* is present in biological fluids, including ascitic fluid, and is capable of forming nests of amastigotes and trypomastigotes in the peritoneal membrane, not only in the chronic phase but also in the acute phase of the infection (40). On the other hand, the histopathological presence of *T. cruzi* amastigotes nests have been described in different organs such as liver, spleen, pancreas or colon; high concentrations of *T. cruzi* DNA was found in the myocardium, urinary bladder, stomach, lymph nodes, adrenal gland, and colon (41–43).

In the present work, we present an analysis of the variation of peritoneal macrophage subpopulations after the intraperitoneal cavity inoculation of mice with EVs of tissue-culture cell-derived trypomastigotes of *T. cruzi*, including analyses of interleukin profiles expressed by these two populations after the EVs-stimulus. Stimulation of mice with immune complexes formed *in vitro* after the incubation of EVs of the parasite with sialylated or non-sialylated IgGs anti-*T. cruzi* are also included, assessing the potential differences that sialylated immunoglobulins could play in the activation processes. In the present work, using as a model of “inflammatory response” the variations in the populations of peritoneal macrophages in the presence of forms of *T. cruzi* already described by (15, 16), we study the role played by the immune complexes formed by the extracellular vesicles of the parasite recognized by sialylated and non-sialylated IgGs, all of them purified from an immunoserum against the parasite antigens, as well as the different expression of cytokines expressed by the different populations of peritoneal macrophages that emerged after intraperitoneal inoculation of the different stimuli.

## 2 Materials and methods

### 2.1 Cell culture and parasite strain

Vero cells (ECACC 84113001) were obtained from the cell bank of the “Centro Instrumentación Científica” of the University of Granada. The cells were cultured in 75 cm^2^ surface area flasks (Thermo Fischer Scientific, Waltham, MA, USA) using Modified Eagle´s Medium (MEM) medium (Sigma Aldrich, St. Louis, MO, USA) supplemented with 10% heat-inactivated (56 °C, 30 min) fetal bovine serum (iFBS) (Gibco, Waltham, MA, USA) plus antibiotics (penicillin 100 U/mL; streptomycin 100 μg/mL) and maintained at 37°C, in a moist atmosphere enriched with 5% CO_2_.

For Vero cell infection with the infective trypomastigote stage of the parasite, or for the collection of its secreted products for EVs purification, the *T. cruzi* Pan4 (Tc Ia + Tc Id) strain (44) was used. This strain was isolated by our group in 2004, in collaboration with Dr. A. Ying of the University of Panama, and it is. maintained in culture and cryopreserved in our laboratory. Cell infections were performed as previously described (34, 45) and trypomastigotes derived from cell cultures were obtained for EVs collection. Briefly, Vero cell monolayers were disrupted with trypsin-EDTA solution, the cells were washed and then seeded on a new culture flask (1 x 10^6^ cells/mL) using MEM + 10% iFBS + antibiotics. Once the cells had attached to the surface of the flask (12 hours), they were washed with sterile phosphate buffered saline (PBS) and subsequently infected with a suspension of the metacyclic forms obtained in culture and purified using Percoll, according to the methodology described by Castanys et al. (46). Cell cultures were incubated with parasites for 6 hours in MEM culture medium (parasite/cell ratio of 3 parasites per cell) and, after the incubation time, non-internalized parasites were removed, cell cultures were washed with PBS, and fresh MEM + 10% iFBS + antibiotics was added. After 96-120 hours of the intracellular development of the parasite, tissue-culture cell-derived trypomastigotes present in culture supernatant were harvested by centrifugation.

### 2.2 Purification of EVs secreted by trypomastigotes

Trypomastigotes were collected from the supernatant of infected cell cultures, in which the parasite had continuously completed a series of intracellular cycles of development. An initial centrifugation of the supernatant at 500 x g for 5 minutes was performed to remove cell debris and, once this centrifugation was completed, the resulting supernatant was centrifuged at 3,000 x g for 15 minutes to concentrate the trypomastigotes in the pellet. Then, the pellet with the parasites was washed 3 times in sterile PBS and 5 x 10^7^ parasites were placed in 75 cm^2^ surface area flasks (Thermo Fischer Scientific, Waltham, MA, USA) with 10 mL of MEM without iFBS (pH 7.2) for EVs secretion. Flasks with the parasites were incubated for 5 hours at 37 °C in 5% CO_2_ enriched atmosphere. Thereafter, the supernatants were collected and centrifuged at 3,500 x g for 15 minutes at 4 °C to remove the parasites.

For EVs isolation and purification, the methodology previously described by our group was employed (34b; 35). Briefly, supernatants with the secreted products of trypomastigotes were subjected to a centrifugation step at 17,000 x g for 30 minutes at 4 °C to remove any apoptotic vesicles and cell debris. After this step, the supernatants obtained were filtered through sterile filters with a pore size of 0.22 µm (Sartorius, Göttingen, Germany, Germany) in order to select EVs with a diameter smaller than the pore size and to remove aggregates or other types of vesicles such as larger ectosomes. The ultrafiltrated samples were then ultracentrifuged at 100,000 x g for 4 hours at 4 °C in a CP100NX ultracentrifuge (Hitachi Koki, Tokyo, Japan) with a P70AT fixed-angle rotor for EVs isolation and, after this centrifugation step, the pellets containing EVs were washed three times by ultracentrifugation using sterile filtered PBS. Finally, EVs were concentrated by ultracentrifugation in a P50A3 fixed-angle rotor and resuspended in 100 µL of sterile filtered PBS.

The isolation procedure and purity of EVs samples were evaluated by transmission electron microscopy, nanoparticle tracking analysis and atomic force microscopy, following the methodologies described in previous works (34–36). The protein concentration of EVs was measured using the microBCA protein assay kit (Thermo Fischer Scientific, Waltham, MA, USA), following the manufacturer’s instructions and the viability of trypomastigotes after the 5-hour period of EVs secretion was assessed by the trypan blue exclusion assay, maintaining a percentage close to 99% of live forms.

### 2.3 Animal handling and permission of the animal welfare and ethics committee

The use of animals was carried out according to institutional guidelines (Spanish Government Regulations: Royal Decree RD1201/05) and European Union guidelines (European Directive 2010/63/EU), having been approved by the Ethics Committee of the University of Granada (Ethics Committee, 235-CEEA-OH-2018), as well as by the authorities of the Regional Government of the Junta de Andalucía with number 12/11/2017/162.

### 2.4 Transmission electron microscopy

For visualization of EVs, samples obtained from the last ultracentrifugation step were resuspended in 30 µL Tris-HCl (pH 7.3) and 5 µL of the suspension was applied directly onto Formvar/carbon-coated grids. After 30 minutes, the grids were washed in PBS and fixed in 1% glutaraldehyde for 30 minutes. After the fixation step, the grids were washed again in PBS and stained and counterstained with 2% (v/v) uranyl acetate. Finally, the samples were observed under a Carl Zeiss SMT LIBRA 120 PLUS TEM microscope. The size of the nanoparticles was measured using the microscope’s own measuring scale and Image J 1.41 software.

### 2.5 Nanoparticle tracking analysis

Distribution, size, and concentration of the EVs samples of *T. cruzi* were determined by measuring the rate of Brownian motion according to the particle size in a Nanosight NS300 (Malvern Instruments, UK). For this purpose, EVs samples were diluted in sterile filtered PBS up to 1 mL and loaded into the high-sensitivity EMCCD chamber for excitation with a 405 nm laser available on the instrument. The Brownian motion images were recorded in three 60-second videos on a camera and analyzed for each of the samples using the instrument’s image analysis software, NTA 2.3 (NanoSight, UK). Measurement conditions were manually adjusted for both shutter, gain, brightness and threshold, all measured at 25 °C. The mean size distribution was calculated as the average of three independent size distributions.

### 2.6 Preparation of polyclonal anti-*T. cruzi* antibodies and separation of sialylated and non-sialylated IgGs

#### 2.6.1 Preparation of polyclonal anti *T. cruzi* antibodies

Five male BALB/c mice were immunized with 20 µg per dose of a total trypomastigote extract of *T. cruzi* Pan4 to produce a polyclonal anti-*T. cruzi* antibody. The parasite extract was obtained from 10^9^ trypomastigotes from cell cultures previously washed and concentrated by centrifugation as described above. The pellet containing the parasites was subjected to three cycles of freezing at -20 °C and slow thawing, and then sonicated in a Branson SLP sonifier (10 s intervals and 10 s pauses, for 2 min). The protein concentration of the lysate was quantified using the microBCA protein assay kit (Thermo Fischer Scientific, Waltham, MA, USA) and aliquoted to 20 µg per 250 µL of PBS.

For the first immunization, the antigen suspension was prepared by mixing it with Freund’s complete adjuvant (Sigma, USA) in a 1:1 ratio (final volume: 500 µL). Freund’s incomplete adjuvant (Sigma, USA) was used for subsequent immunizations up to a total of 7 (one per week). The antibody titers of the serum samples were determined weekly after the first two immunizations by indirect ELISA and, at the end of the immunization period (8 weeks) mice were euthanized under isoflurane. Whole blood samples were obtained by cardiac puncture and sera were collected using BD Microtainers^®^ (BD, Franklin Lakes, New Jersey, USA). Final antibody titers were determined by enzyme-linked immunosorbent assay (ELISA) as described elsewhere.

#### 2.6.2 Electrophoretic separation of proteins and Western blot

Proteins from EVs samples of *T. cruzi* were precipitated in acetone at -20 °C overnight. The precipitated samples were centrifuged at 13,000 x g for 10 minutes at 4 °C and washed twice with cold acetone. Finally, the acetone was evaporated under a nitrogen stream and the precipitated proteins were quantified using the MicroBCA protein assay kit (Thermo Fischer Scientific, Waltham, MA, USA). For electrophoresis, 30 µg of proteins of EVs were loaded onto 12% SDS-PAGE gels and then transferred to PVDF membranes (BioRad, Hercules, CA, USA) in a Turbo Trans-Blot transfer system (BioRad, Hercules, CA, USA). The membranes were immersed in blocking buffer (PBS, 0.1% Tween 20 and 4% nonfat dry milk) and incubated for 2 hours at 4 °C and under gentle shaking. The blocked membranes were then incubated with a 1:1,000 dilution of the polyclonal anti-*T. cruzi* antibodies overnight at 4 °C. After this incubation, the membranes were washed and incubated with peroxidase-conjugated goat anti-mouse IgG (1: 1,000) (Dako Agilent Pathology Solutions, USA) for 1 hour at room temperature. The detected bands were visualized using Clarity ECL Western Substrate (BioRad, Hercules, CA, USA) on a ChemiDoc Imaging system (BioRad, Hercules, CA, USA).

#### 2.6.3 Purification of sialylated and non-sialylated IgGs

IgGs anti-*T. cruzi* were purified from polyclonal anti-*T. cruzi* antibodies using Melon gel chromatography gel monoclonal IgG purification kit (Thermo Fischer Scientific, Waltham, MA, USA), following the manufacturer’s instructions. Microcolumns were prepared by placing 500 μL of gel in an empty cartridge that was centrifuged at 3,000 x g for 1 minute to densify the gel, followed by two washes with 300 μL of the purification buffer provided with the kit. Then, 0.5 mL of serum with polyclonal antibodies were diluted 1:10 with the purification buffer, added to the column and incubated for 5 minutes under agitation. The column was then centrifuged to collect the purified IgGs present in the elution.

In order to separate sialylated from non-sialylated IgGs, an affinity chromatography using minicolumns coupled to Sambucus nigra lectin (SNA, EBL) agarose (Vector Laboratories, Newark, CA, USA) was performed. For this purpose, 500 μL of Sambucus nigra lectin (SNA, EBL)-agarose were placed in an empty cartridge (Thermo Fischer Scientific, Waltham, MA, USA) and submitted to a centrifugation step at 3,000 x g for 1 minute to densify the matrix of these columns. Then, 3 washing steps of the column with PBS, followed by 3 washing steps with Hank`s solution (Sigma Aldrich, St. Louis, MO, USA) were performed. IgGs previously purified using the Melon Gel Chromatography Purification Kit were diluted in Hank’s solution at a 1:1 ratio and added to the lectin for 10 minutes at room temperature with gentle agitation. Thereafter, the column was centrifuged at 3,000 x g for 1 minute and the eluate (containing non-sialylated IgGs) was collected. The column was washed 4 times with PBS, collecting the eluate after each step.

Elution of sialylated IgGs was performed by adding 2 volumes of 0.5 M lactose in PBS, followed by further washing with 2 volumes of 0.5 M lactose and 0.2 M acetic acid in PBS; once the sialylated IgGs was eluted, the pH of the samples was neutralized to 7.2 by adding Tris base. Both sialylated and non-sialylated IgGs were dialyzed against 0.1 M ammonium acetate for 24 hours with at least 4 changes of the dialysis fluid. The protein concentration of the samples was quantified using the microBCA protein assay kit (Thermo Fischer Scientific, Waltham, MA, USA) and after quantification, the samples were aliquoted and lyophilized. Before use, lyophilized IgGs were resuspended in sterile PBS. Both the purity of the samples and whether or not they were sialylated IgGs were assessed by 12.5% SDS-PAGE electrophoresis followed by Western blot.

**Figure 1:**
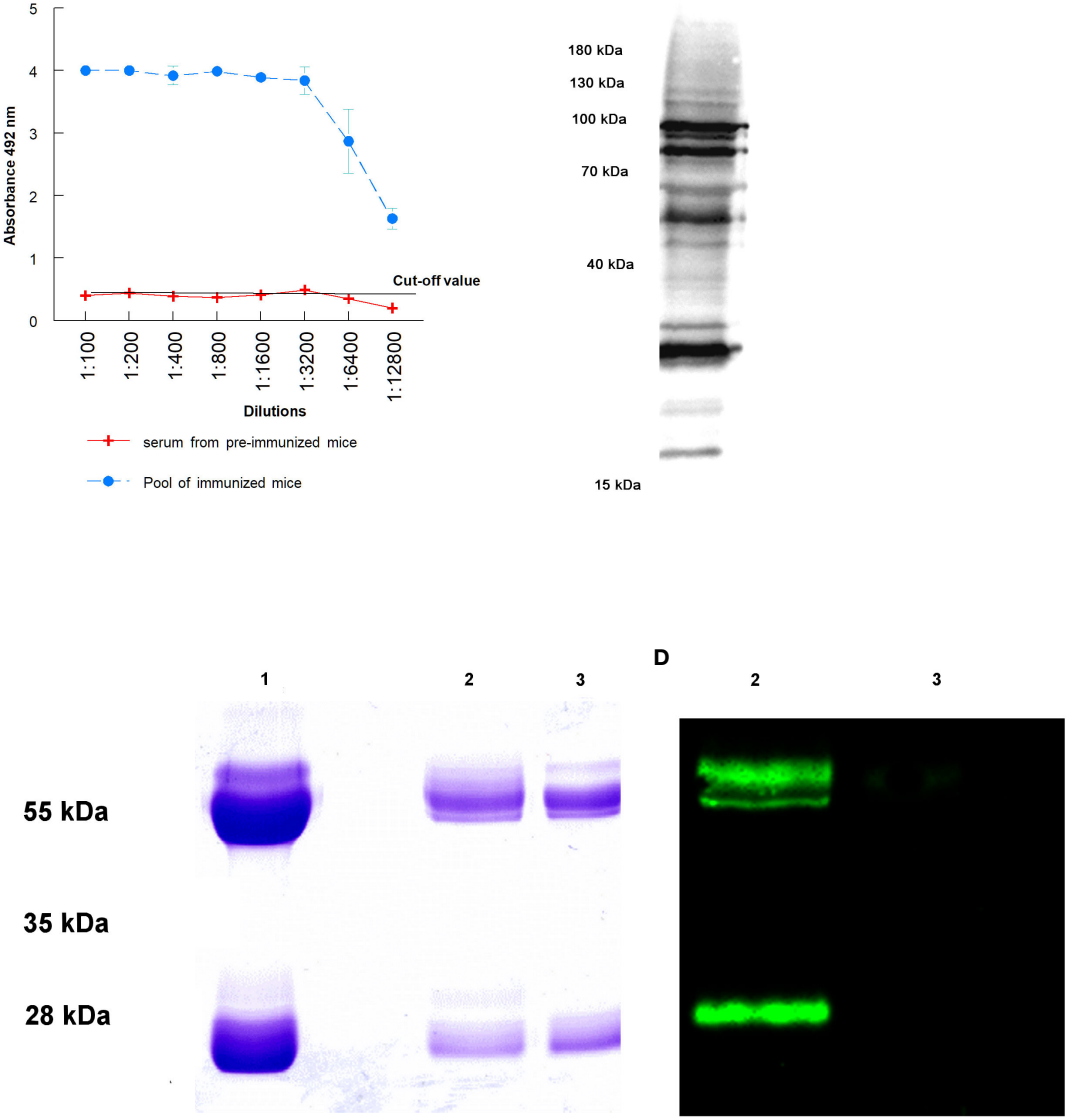
Purification of IgGs anti *T. cruzi* after the immunization of mice: **(A)** antibody titers obtained against total proteins of trypomastigotes of *T. cruzi*; **(B)** antibody recognition of total proteins from a lysate of trypomastigotes by Western blot; **(C)** SDS-PAGE electrophoresis of purified immunoglobulins: purification of total IgGs by Melon gel chromatography (1), sialylated (2) and non-sialylated IgGs (3) after affinity chromatography using Sambucus nigra lectin; **(D)** Western blot analysis using biotinilated Sambucus nigra lectin and revealed with streptavidin HRP: sialylated (2) and non-sialylated IgGs (3).

For Western blot, 10 µg of sialylated and non-sialylated IgGs obtained as previously described were separated by 12.5% SDS-PAGE gel electrophoresis. After electrophoretic separation, transference to PVDF membranes (BioRad, Hercules, CA, USA) was performed using a Trans-Blot turbo transfer system (BioRad, Hercules, CA, USA). The membranes were then blocked and washed as previously described, and incubation with 5 μg/mL Sambucus nigra biotin-linked lectin (SNA, EBL) (Vector Laboratories, Newark, CA, USA) for 25 minutes at room temperature was performed. After this incubation, the membranes were washed 6 times in PBS-Tween 20 0.1% and then incubated with streptavidin-HRP (1:8000) for 45 minutes at room temperature. Finally, the membranes were washed 6 times with PBS-Tween 20 0.1% and visualized on a ChemiDoc MP imaging system (BioRad, Hercules, CA, USA) (**Figure 1C**).

#### 2.6.4 Recognition of EVs proteins by IgGs anti-*T. cruzi*

To evaluate the recognition and binding of proteins in EVs by polyclonal anti-*T. cruzi* IgGs, 30 µg of EVs were resolved by SDS-PAGE electrophoresis and analyzed by Western blot, as previously described. For this purpose, the analyses were performed using the anti-*T. cruzi* IgGs separated from the polyclonal anti-*T. cruzi* antibodies produced in mice (1:100). Goat anti-mouse IgGs conjugated with peroxidase (Agilent Technologies, Santa Clara, CA, USA) (1: 1,000) were used as secondary antibodies and visualization was performed in a ChemiDoc MP imaging system (BioRad, Hercules, CA, USA).

#### 2.6.5 Preparation of immune complexes (ICs) EVs - IgGs anti-*T. cruzi* and inoculation of mice

To carry out the formation of immune complexes *in vitro* using EVs of trypomastigotes and IgGs anti-*T. cruzi*, a methodology previously described was applied, and the amount of proteins of EVs necessary to carry out the experiment was set at a rate of 38 µg of EVs per mouse (equivalent to approximately 5.087 x 10^8^ EVs) (35).

**Figure 2:**
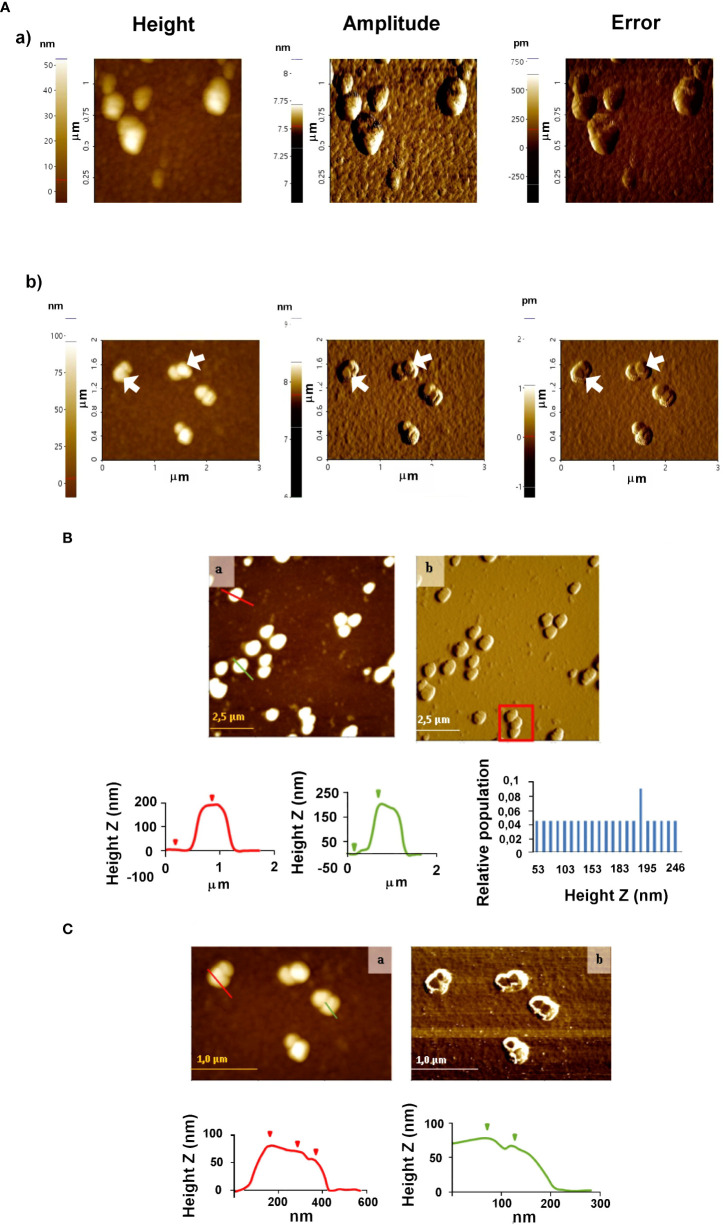
Atomic force microscopy analyses of EVs secreted by trypomastigotes of *T. cruzi* **(A)** and **(B)** immune complexes EVs-IgGs anti *T. cruzi* **(A)**. Non-contact **(B)** and tapping **(C)** modes were employed for the analysis of ICs. In C, the formation of ICs as a result of the binding of IgGs to EVs is evidenced by changes in the height profile as a result of that binding. Graphs show profile lines that correspond to topography images, in which changes in the profile are observed as changes in the heights of the particles identified (for the red profile: first step of 8.167 nm and second step of 10.082 nm in height; for the green profile: Z height of 11.220 nm).

Incubation of EVs and anti-*T. cruzi* IgGs (sialylated and non-sialylated IgGs) was performed under orbital shaking (10 rotations per minute) at 37 °C for 1 hour. Thereafter, the samples were washed 3 times by ultracentrifugation in sterile-filtered PBS plus protease inhibitors (Roche, Switzerland) to remove unbound immunoglobulins and each pellet was then resuspended in 100 µL of sterile-filtered PBS and stored at 4 °C until inoculation into the animals. An aliquot of the immune complexes obtained was examined by atomic force microscopy (**Figure 2**).

For the intraperitoneal inoculation of mice with the previously prepared stimuli, 4 animals were used for each batch, which consisted of 250 µL of PBS containing an equivalent amount of proteins from each of the different fractions of the purified IgGs (sialylated or non-sialylated IgGs).

### 2.7 Atomic force microscopy

To visualize the binding of the anti-*T. cruzi* IgGs to the purified EVs released by trypomastigotes, atomic force microscopy analyses were performed, following the methodology previously described (36). For these experiments, 8 µL of the suspension of ICs formed *in vitro* (EVs-IgGs) were deposited onto the moscovite mica slide that served as the substrate, and the samples were incubated for 15 minutes. Then, 3 washing steps with milliQ ultrapure water (Millipore, Burlington, MA, USA) were performed and the samples were dried under a gentle stream of argon prior to the visualization.

Atomic force microscopy analysis was performed using non-contact and tapping modes, in NX-20 equipment (Park Systems, Suwon, Korea) and images were acquired and processed as described by Retana Moreira et al. (36), using the XEI software (Park Systems, Suwon, Korea). Samples of purified EVs were also analyzed.

### 2.8 Flow cytometry analysis for macrophage separation

For these experiments, six-week-old female C57BL/6 wild-type mice maintained under pathogen-free conditions were used and six different groups were established and inoculated with a final volume of 250 µL of the following preparations: 1) EVs (38 µg/mouse), 2) immune complexes EVs - sialylated IgGs, 3) immune complexes EVs - non-sialylated IgGs, 4) trypomastigotes derived from cell cultures and resuspended in PBS (10^6^ parasites), and 5) LPS (10 µg). The last two preparations were included as positive controls, as described previously (16). A final batch of mice inoculated with 250 µL of PBS was also included as negative control. All inoculations were performed intraperitoneally in a final volume of 250 µL in PBS.

After 48 h of stimulation of the mice with the preparations previously described, the mice were anesthetized in isoflurane atmosphere, euthanized by cervical dislocation and peritoneal exudate cells (PEC) were collected by washing the peritoneal cavity of each animal with 5 mL of a cold 0.85 mM EDTA solution in sterile PBS. Peritoneal cells were centrifuged at 1,000 x g for 10 minutes at 4 °C and the pellets obtained from centrifugation were resuspended in ACK lysis buffer (0.15M NH_4_Cl, 0.01M KHCO_3_, 0.1mM Na_2_EDTA, pH 7.2), where they were held for 5 minutes to lyse red blood cells contaminating the peritoneal exudate. After this lysis step, the cells were centrifuged at 1,000 x g for 5 minutes and the pellets were resuspended in 200 µL of PBS.

The cell suspensions were incubated for 20 minutes in the dark at room temperature with the labeled monoclonal antibodies included in **Table S1**. Incubations with each antibody were performed in separate and after each incubation, the cells were centrifuged at 1,000 x g for 5 minutes, unbound antibodies were removed and two washes with 500 µL sterile PBS were performed. With this panel of antibodies, the frequency of two different subpopulations of peritoneal macrophages was measured and a sorting separation of these populations into large peritoneal macrophages or LPM (CD19-, CD11c-, CD11bhi, F4/80hi and MHCIIlo) and small peritoneal macrophages or SPM (CD19-, CD11c-, CD11blo, F4/80lo and MHCIIhi) was carried out (15, 18). Briefly, for this separation and after the incubation with all the antibodies of the panel, singlets were chosen. Then, viable. B cells were removed, selecting CD19- cells. Dendritic cells were also removed gating CD11c- cells and finally, LPM and SPM were selected according to the expression levels of CD11b and F4/80.

### 2.9 Cytokine expression analyses in large and small peritoneal macrophages

After the separation and purification of both macrophage subpopulations, the cell suspensions were centrifuged at 1,000 x g for 10 minutes and the cell pellets were lysed and homogenized with TRIzol reagent (Thermo Fischer Scientific, Waltham, MA, USA) for RNA purification. After the extractions, incubation with DNase I, RNase-free (Thermo Fischer Scientific, Waltham, MA, USA) was performed according to the manufacturer’s recommendations, and the absence of gDNA was confirmed by the absence of a positive amplicon in PCR amplification for the actin gene.

Once the RNAs were purified and quantified, the expression of cytokines and markers (*IL-1β, IL-2, IL-12, IL-18, TNF-α, IFN-γ, G-CSF, IL-6, IL-15, IL-10, TGF-β, IL-17, IL-38*, and nitric oxide synthase (*NOS*) was analyzed by RT-qPCR, using the iTaq™ universal SYBR^®^ Green one-step universal SYBR^®^ kit (BioRad, Hercules, CA, USA), employing *gapdh* as the reference gene. The sequences of the primers employed in this analysis are listed in **Table S2**; primer sequences were located across exon–exon borders, avoiding any interspecifically and intraspecifically variable positions. Moreover, a calibration curve was performed according to Gomez-Samblas et al. (47) to calculate the efficiency of each pair of primers,.

Reactions were performed in a CFX-96 RT-PCR system (BioRad, Hercules, CA, USA), using a final volume of 10 µL, which included 300 nM of each primer and 100 ng of RNA per reaction. The thermal cycling conditions consisted of retrotranscription at 50 °C for 10 minutes, followed by an enzymatic activation step and DNA denaturation at 95 °C for 1 minute, 40 cycles of denaturation at 95 °C for 10 seconds and an annealing and extension step at 60 °C for 30 seconds, followed by plate reading. At the end of the RT-qPCR reactions, a melting gradient was applied from 65 °C to 95 °C in 0.5 °C increments. Cytokine expression was also normalized against *gapdh* and the negative control (PBS-injected mice).

## 3 Results

### 3.1 Isolation and characterization of EVs

Extracellular vesicles of trypomastigotes of *T. cruzi* were obtained following a sequential process of differential centrifugation, filtration and ultracentrifugation, a method previously employed by our research group (35, 36). EVs isolation and characterization was performed following the Minimal Information for the Study of Extracellular Vesicles (MISEV) guidelines (28), recommended by the International Society for Extracellular Vesicles (ISEV) for all articles involving such vesicles. Transmission electron microscopy of the samples observed by negative staining, nanoparticle tracking analysis and atomic force confirmed the presence of vesicles of a size compatible with exosomes and smaller ectosomes of the infecting stage of the parasite, described by our group in previous publications (36) (**Figure S1**). In particular, TEM revealed a mean size of vesicles of 46.3 nm (median: 45.7 nm) (**Figure S1A**) and majority peaks of vesicles corresponding to 42 and 52 nm was observed using NTA (**Figure S1B**). In all of the analyses, aggregations of EVs were observed, especially in TEM and AFM (**Figures S1A, C**).

Western blot analysis using an antibody against cruzipain revealed the presence of bands of 35 kDa – 70 kDa, corresponding to this *T. cruzi*-specific enzyme in EVs (**Figure S1D**).

### 3.2 Purification of sialylated and non-sialylated IgGs and formation of immune complexes EVs – IgGs

The success in mice immunization with a total extract of trypomastigotes of *T. cruzi* and the consequent purification IgGs anti-*T. cruzi* from sera from infected mice is presented in **Figure 1**. **Figure 1A** shows antibody titers obtained by immunization of mice with the total extract of trypomastigotes obtained from cell cultures. From the ELISA, the 1: 12,800 dilution of the hyperimmune serum revealed an absorbance 3 times higher than that the titer of sera from non-immunized mice, in which the cut-off value was obtained. **Figure 1B** shows the antigenic recognition of antigens of *T. cruzi* in the total extract of trypomastigotes by sera of immunized mice using Western blot.

**Figure 1C** the Lane 1 shows the result of the purification of total IgGs from sera using Melon gel chromatography. In this Figure, bands corresponding to the heavy and light chains of the purified IgGs are observed. Moreover, affinity chromatography with Sambucus nigra lectin was performed to separate sialylated from non-sialylated IgGs; results from this process are shown in **Figure 1C** (2 and 3 lanes. In this Figure, lane 2 corresponds to sialylated-IgGs, which were retained and subsequently eluted with 0.5 M lactose, while lane 3 corresponds to non-sialylated-IgGs that were not retained in the previous affinity chromatography. **Figure 1D** shows a Western blot that confirms the proper purification of sialylated and non-sialylated IgGs, following the protocol described in Material and Methods section.

AFM was also employed to study the morphology and distribution of *T. cruzi* EVs and those with which immune complexes were formed by incubating purified IgGs from anti-*T. cruzi* sera with the purified EVs. Once the ICs resulting from this incubation were formed, they were purified and washed by ultracentrifugation, and non-covalently immobilized on the mica sheet for subsequent observation by AFM. The results obtained are shown in **Figure 2**, which also confirmed the presence and integrity of the vesicles. For EVs, homogeneous particles of 20-50 nm were observed (**Figure 2A**). In the case of the immune complexes (**Figures 2A, B**), larger structures were observed both in the non-contact (**Figure 2B**) and tapping modes (**Figure 2C**), suggesting that they could correspond to more than one IgGs aggregated with one or more different EVs, as length values between 0.8 µm and 1.5 µm were obtained (**Figure 2B**, red square); however, length values from 0.35 to 1.35 µm and height values from 50 to 250 nm prevailed. **Figure 2C** shows immune complexes of different globular shapes and sizes, with length values from 0.175 to 1.8 µm and height values from 50 to 246 nm. In the same **Figure 2C**, changes in the profile lines are evident, which can be interpreted as changes in the heights of the identified particles.

### 3.3 Flow cytometry analysis for macrophage separation

**Figures S6**-**S11** show the separation of cell subpopulations in the peritoneal cavity of mice stimulated with different preparations using sorter flow cytometry. Results include both the number of events and the percentages of cell subpopulations 48 hours after the inoculation with the samples included in this study: EVs, immune complexes formed by the incubation of EVs of *T. cruzi* with sialylated or non-sialylated IgGs, LPS and trypomastigotes of *T. cruzi* (positive controls) and PBS (negative control). Results regarding the number of cells obtained for each stimulation are shown in **Table S3**. The same figures (**Figures S6**-**S11**) also show how cell subpopulations were selected using antibodies and flow cytometry.

**Figure 3:**
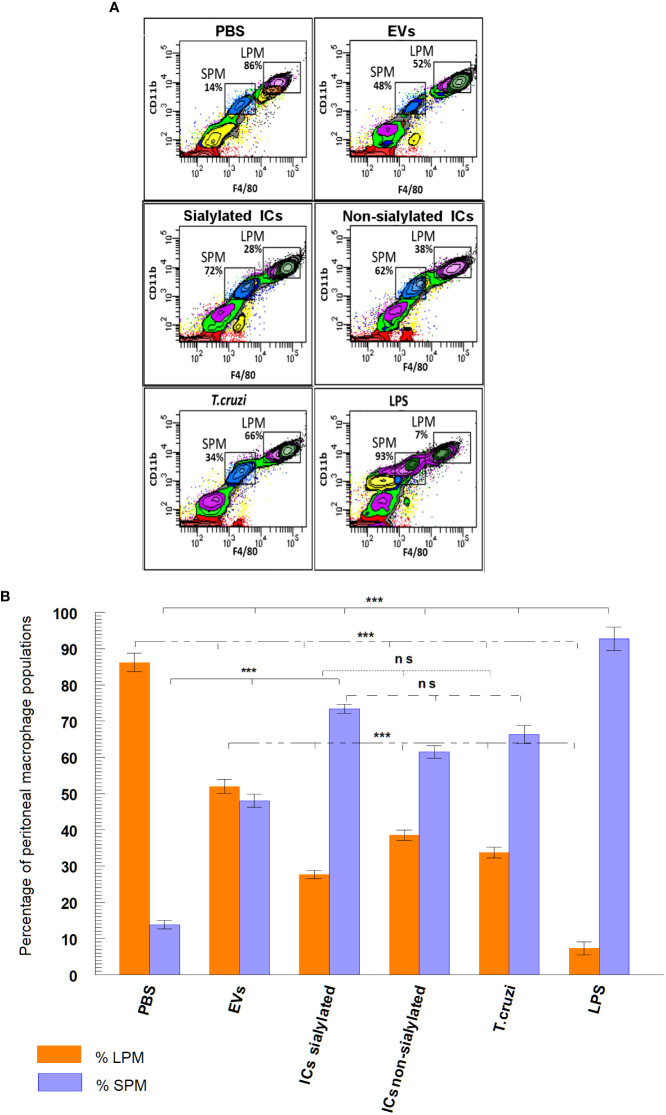
Separation **(A)** and percentage **(B)** of macrophage subpopulations (small and large peritoneal macrophages) collected from the peritoneal cavity of mice after different stimuli using flow cytometry: PBS, EVs (extracellular vesicles shed by trypomastigotes of *T. cruzi*), sialylated ICs (EVs - IgGs anti *T. cruzi* retained after affinity chromatography using Sambucus nigra lectin), non-sialylated ICs (EVs - IgGs not retained after affinity chromatography using Sambucus nigra lectin), *T. cruzi* (trypomastigotes) and LPS. The statistical test used was the Tukey-Kramer Multiple Comparisons Test (*** = *p* < 0.001).

**Figure 3A** shows the final plots of the different macrophage subpopulations recovered from the peritoneal cavity of the mice, 48 hours after the stimulation with the samples described above. In **Figure 3B**, macrophage subpopulations recovered after mice stimulation with the samples, as well as the statistical significance of the difference in the numbers of small and large peritoneal macrophages are presented in a bar graph. Results show that in the peritoneal cavity of mice inoculated with PBS (negative control), there is a larger population of LPM (86.2%) compared to 13.8% of SPM, while for LPS (positive control), the percentage of LPM is drastically reduced to 7.3% and SPM increased to 92.7%. When mice were stimulated with trypomastigotes of *T. cruzi*, the percentage of LPM decreased to 33.7% as compared to the negative control, while SPM reached 66.3%. However, when mice were stimulated with EVs secreted by this stage of the parasite, percentages of LPM and SPM tended to equalize (52% for LPM and 48% for SPM). A percentage of LPM significantly reduced with respect to the percentage of LPM obtained when mice were inoculated with immunoglobulin-free EVs (27.6%); for SPM, the percentage of cells reached 73.4% of the total population of macrophages. Finally, mice stimulated with ICs formed by the incubation of EVs with non-sialylated IgGs reached macrophage percentages of 38.5% for LPM and 61.5% for SPM.

### 3.4 Cytokine expression analyses in large and small peritoneal macrophages stimulated with EVs and immune complexes EVs - IgGs anti-*T. cruzi*

**Figure 4:**
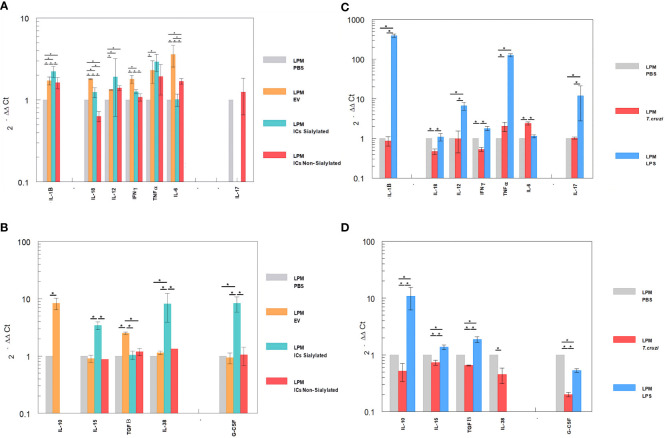
Relative interleukin expression of the LPM subpopulation after the stimuli of mice with EVs of *T. cruzi*, as well as sialylated and non-sialylated ICs (EVs – IgGs anti-*T. cruzi)* versus controls stimulated with trypomastigote forms of the parasite or LPS. **(A)**, relative expression levels of inflammatory interleukins produced by LPM after stimulation with EVs or immune complexes formed by sialylated or non-sialylated IgGs. **(B)** shows the expression levels of non-inflammatory interleukins produced by LPM after stimulation with EVs or ICs formed by sialylated or non-sialylated IgGs. **(C)** shows the expression levels of inflammatory interleukins produced by LPM after stimulation with trypomastigote forms of the parasite or with LPS (stimulation controls). **(D)** shows the expression levels of non-inflammatory interleukins produced by sorter-purified LPM after stimulation of the peritoneal cavity with *T. cruzi* forms or with LPS (stimulation controls). The statistical test used was the Tukey-Kramer Multiple Comparisons Test. (*= *p* < 0.05).

**Figure 5:**
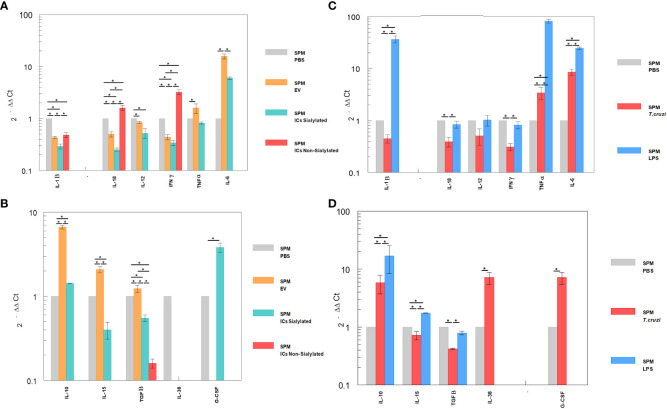
Relative interleukin expression by SPM subpopulation after the stimuli of mice with EVs of *T. cruzi*, as well as sialylated and non-sialylated ICs (EVs – IgGs anti-*T. cruzi*) versus control stimulated with trypomastigote forms of the parasite or LPS. **(A)**, relative expression levels of inflammatory interleukins produced by SPM after stimulation with EVs or immune complexes formed by sialylated or non-sialylated IgGs. **(B)** shows the expression levels of non-inflammatory interleukins produced by SPM after stimulation with EVs or ICs formed by sialylated or non-sialylated IgGs. **(C)** shows the expression levels of inflammatory interleukins produced by SPM after stimulation with trypomastigote forms of the parasite or with LPS (stimulation controls). **(D)** shows the expression levels of non-inflammatory interleukins produced by sorter-purified SPM after stimulation of the peritoneal cavity with *T. cruzi* forms or with LPS (stimulation control). The statistical test used was the Tukey-Kramer Multiple Comparisons Test. (*= *p* < 0.05).

The differential expression of interleukins by the LPM and SPM populations, recovered from the peritoneal cavity of mice after the 48-hour stimulation with EVs and ICs and separated by the sorter, is shown in **Figures 4**, **5A**. **Figures 4A, B** shows the cytokine expression in LPM after the stimulation with EVs or after forming immune complexes with sialylated or non-sialylated IgGs. In, **Figures 4C, D** shows the expression levels of the non-inflammatory interleukins in peritoneal macrophages after the stimulation with trypomastigotes of *T. cruzi* or LPS (positive controls). The expression of Th1 inflammatory interleukins reflects how, in general, the stimulation with EVs induces a higher expression of IFN-γ, TNF-α, IL-18 and IL-6. As can be observed in **Figure 4A**, there is an increase in the IL-1β expression in macrophages from the 3 conditions in which EVs of *T. cruzi* were used. This is followed by the stimulation produced by the immune complexes formed using sialylated IgGs, where an increased expression of IL-12 and TNF-α is induced. Moreover, the IFN-γ, IL1B and IL12 interleukins were upregulated when the peritoneal cavity of mice was stimulated by LPS (**Figure 4C**). For the case of trypomastigotes, expression of IL-6 and IL-18 resulted higher when compared to PBS control. Regarding IL-17 expression, LPM showed an increased expression level after stimulation with LPS, **Figure 4C**.

**Figures 5A, B** shows the relative expression levels of inflammatory and non-inflammatory interleukins produced by SPM after stimulation with EVs or immune complexes formed by sialylated or non-sialylated IgGs. **Figure 5C** shows the expression levels of inflammatory interleukins produced by SPM after stimulation with trypomastigote forms of the parasite or with LPS. **Figure 5D** shows the expression levels of non-inflammatory interleukins produced by sorter-purified SPM after stimulation of the peritoneal cavity with *T. cruzi* forms or with LPS (stimulation control). For IL-10, the highest level of expression occurred after the injection of the peritoneal cavity with EVs and in stimulation control experiments with LPS (**Figures 5B, D**, respectively). Other cytokines such as IL-15, IL-38 or G-CSF, also presented maximum expression levels after the stimulation with immune complexes formed with sialylated IgGs.

The SPM subpopulation underwent significant variations after the different stimuli (**Figure 5**), either with the ICs or after the challenge with trypomastigotes or LPS. In this sense, the highest expression levels of IFN-γ were obtained after the stimulation of these cells with the immune complexes with non-sialylated IgGs, while stimulation with EVs of the parasite induced maximal expression levels of TNF-α, IL-6 (**Figures 5A, C**), IL-10, IL-15 and TGF-β (**Figures 5B, D**), the stimulation with immune complexes with sialylated IgGs induced increased expression of both the inflammatory interleukin IL-6 (**Figure 5A**) and the regulatory interleukins IL-10 and G-CSF (**Figure 5B**). For the latter, expression was not induced by either EVs or non-sialylated immune complexes, and in the case of control experiments, expression of G-CSF was only achieved when the stimulation was performed with trypomastigotes of *T. cruzi* (**Figure 5D**). The SPM did not express IL-17 in any case, and expression levels for interleukin IL-38 were obtained only when the stimulation was performed in the control experiment after the inoculation with trypomastigotes (**Figure 5D**).

**Figure 6:**
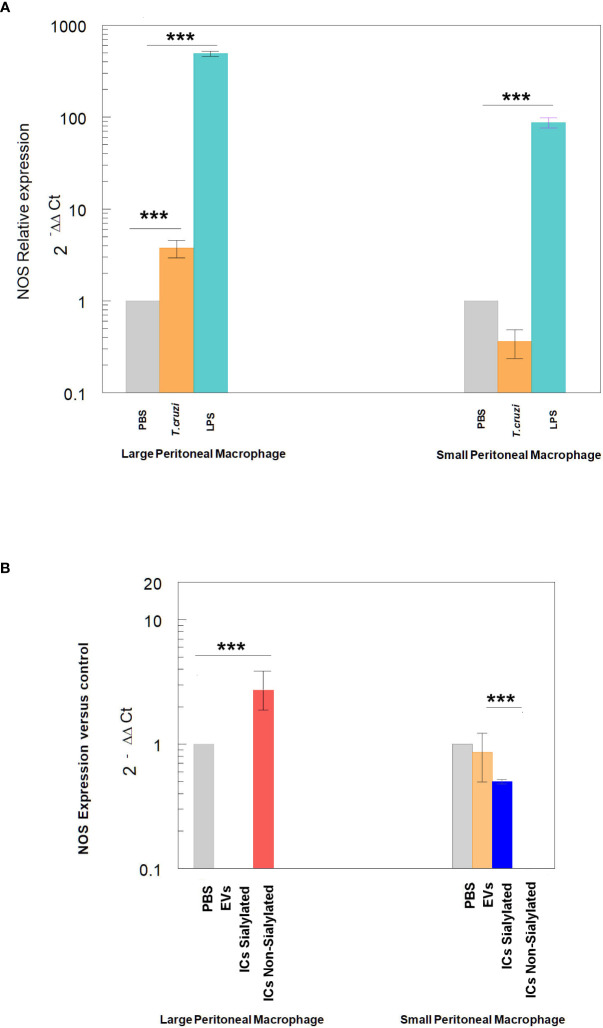
Relative expression of nitric oxide synthase (NOS) in SPM and LPM subpopulations after the stimuli of mice with: trypomastigotes of *T. cruzi* and LPS **(A)**, and EVs of *T. cruzi* and sialylated or non-sialylated ICs. **(B)** Relative expression of nitric oxide synthase (NOS) in LPM and SPM peritoneal macrophages. The statistical test used was the Tukey-Kramer Multiple Comparisons Test. (*** = *p* < 0.001).

Finally, the expression of NOS by large and small peritoneal macrophages of the control treatments is shown in **Figure 6**, where the stimulation with LPS or trypomastigotes of *T. cruzi* induces the expression of NOS in LPM at a higher level than SPM, which even showed lower expression levels than macrophages obtained after the injection of mice with PBS. Injection of either EVs or the two types of immune complexes in SPM did not induce NOS expression levels higher than the PBS control under our experimental conditions.

## 4 Discussion

Macrophages are considered to be part of the first barrier to prevent infections, acting as antigen-presenting cells to CD4+ T lymphocytes or as phagocytic cells of previously opsonized antigens. During an infection, monocytes/macrophages acquire the M1 phenotype (IL-10-, IL-12+, NOS+, CXCL9+), which are the major effector cells for the first line of host antibacterial defense (48). On the other hand, M2 macrophages refer to alternatively activated macrophages and can be polarized by various stimulatory factors; moreover, different M2 macrophage subtypes can be induced by different stimulatory factors (49). In 2008, Mosser and Edwards proposed that the classification of macrophages could be based on the functions in which they participate, including host defense, wound healing, and immune regulation (50). From subtypes of M2 macrophages, M2a (also referred to as wound healing macrophages) are induced by IL-4 and IL-13, and express high levels of mannose receptor (MR, also referred to as CD206), IL-1 decoy receptor (IL-R) and CCL17, which promote the secretion of pro-fibrotic factors such as TGF-β, insulin-like growth factor (IGF) and fibronectin to contribute to tissue repair (48). On the other hand, bacteria, viruses, and some protozoan parasites like Leishmania can increase the number and proportion of M2b macrophages in the peritoneal cavity (IL-10+, IL-12-, IL-6+, TNF-α+, CD11b+, MHCII+) (51, 52), which, despite their high phagocytic capacity, are not the main cells responsible for killing bacteria (53).

The secretion of EVs by trypomastigotes of *T. cruzi* favors the invasion process to the host cell and modulates the immune response so that parasitization can be successful. In a recent publication, Cortes Serra et al. (54) review and discuss the role of EVs secreted during a *T. cruzi* infection and their immunomodulatory properties (54). It is also well known that EVs of the parasite induce physiological changes, including increases in the intracellular calcium concentration and modifications at the cytoskeletal level such as actin depolymerization (35), which could maybe alter macrophage migration and their phagocytic capacity. Moreover, EVs released by THP-1 cells that are in contact with the parasite are also able to modulate the immune response of the host by inhibiting C3 convertase; complement system inhibition is also achieved directly by EVs released by the parasite (34b).

The role of EVs of *T. cruzi* in inducing the release of pro-inflammatory cytokines (TNF-α and IL-6) *in vivo*, as well as the production of NO by murine macrophages after the interaction with TLR2 has been previously reported (38, 39). Moreover, in 2018, Lovo Martins et al. revealed that macrophages from the bone marrow stimulated with EVs of the parasite increased prostaglandin E2 (PGE2) production and the formation of lipid bodies; the increase in PGE2 has been related to an augmented susceptibility of the host to *T. cruzi* invasion (55, 56). Moreover, PGE2 can also affect the antibody production by blocking the transformation of B lymphocytes into plasmatic cells, and inhibit the role of T cytotoxic cells and Th1 lymphocyte development (57). The ability of EVs of the parasite in inducing the release of proinflammatory cytokines has also been confirmed using *in vitro* models, in which the secretion of nitric oxide (NO) by THP-1 macrophages was also confirmed (39). Besides, Nogueira et al. (38) analyzed the immune response induced by these vesicles in peritoneal macrophages from C57BL/6 mice and their results coincide with increased expression levels of TNF-α and IL-6 in LPM of our study. On the other hand, it has also been described how the stimulation of animals with EVs of *T. cruzi* prior to the infection with trypomastigotes induced a decrease in NO production in plasma, as well as the production of TNF-α and IL-6 in spleen cells, but an increase in IL-4 and IL-10 in splenocytes and macrophages that induced high parasitemia and the death of the mice. It was confirmed in this study that peritoneal cells also synthetize IL-12, TNF-α and NO after EVs stimulation (56, 58).

Extracellular vesicles released by *T. cruzi* contain a repertoire of key components that are also capable of stimulating TLR (59) as it happens with the whole parasite. The cargo of these EVs includes nucleic acids, proteins with protease activity like GP63 and serine proteases, and GPI-anchored members of the large mucin family that include *trans*-sialidases, all of them capable of activating TLR2 and TLR9 and induce a pro-inflammatory response (60). The role of *trans*-sialidases in EVs over cell physiology and the modulation of the immune response has also been studied by our research group, and it is likely that these enzymes, capable of transferring sialic acid from the host cell to mucins of the parasite might be responsible, at least in part, for lymphoid cell modulation (36).

In this work, the exposure of extracellular vesicles of trypomastigotes of *T. cruzi* to the immune system could be considered an approach to the early stage of the infection (acute phase of the disease), while the injection of immune complexes EVs - IgGs anti *T. cruzi* could simulate the chronic stage, in which most of the circulating anti *T. cruzi* immunoglobulins are IgGs which must participate in the formation of these complexes, as previously reported by our group (34a). Results obtained under our experimental conditions show that the stimulation of C57BL/6 mice with trypomastigotes of the parasite and LPS triggered an increase in the proportion of SPM when compared to the negative control (PBS-stimulated mice) (**Figure 3B**); similar results as those obtained by 18 (18). When the stimulation was performed with EVs of the parasite, an increase in the proportion of SPM was also observed, but in a minor proportion as for trypomastigotes or LPS. However, when mice were challenged with both types of immune complexes (sialylated and non-sialylated ICs), the proportion of SPM was significantly higher than when the challenge was performed with EVs, which could suggest that even when EVs are an immunologic stimulus capable of increasing the SPM population, surface components of the vesicles that are not blocked by the Fab region of IgGs anti-*T. cruzi* are likely to suppress the stimulatory response in lymphoid cells of the peritoneal cavity of mice, which would impede the recruitment of circulating monocytes to the peritoneal cavity.

Differential expression levels of cytokines in mice stimulated with EVs or with the immune complexes EVs - IgGs anti *T. cruzi* were found, which could be related to the fact that IgGs present in these ICs interact with Fcγ receptors. IgGs bind to specific Fc receptors present on the cell membranes of macrophages and allow the opsonization of antigens and phagocytosis of immune complexes. Based on these receptors present on the surface of the cells, the effector functions initiated by antibodies are triggered by the Fc domain upon binding to FcR receptors, functions that are largely dependent on the N-terminally bound biantennary glycan of immunoglobulin heavy chains, located below the immunoglobulin hinge region. This glycan is considered to hold the two Fc heavy chains in an open conformation necessary to interact with activating Fcγ receptors (FcγRs). However, the presence in the sugar chain of terminal sialic acid in the glycan has profound implications on the effector functions of Fc by inhibiting activation by cells (22), as FcRγIIB receptors would be responsible for binding to such immunoglobulins carrying sialic acid in their Fc (23). In this sense, the presence of sialic acid, bound in an α2,6 bond to the penultimate terminal galactose of the glycan (24) reduces FcγR binding and converts IgGs antibodies to anti-inflammatory mediators (25), thus sialylated IgGs would be responsible for the anti-inflammatory activity of intravenous immunoglobulin (IVIg) therapy (25). IVIg therapy is based on the anti-inflammatory activity of immunoglobulins derived from plasma of healthy donors and is commonly employed in inflammatory pathologies such as autoimmune diseases (61) due to its regulatory properties, as it induces a polarization of M2 into M1 macrophages (especially in tumor-related macrophages). For this therapy, immune complexes with sialylated Fcs initiate an anti-inflammatory cascade *via* the receptor or lectin SIGN-R1 or DC-SIGN (26), which works as a receptor of IgGs enriched in sialic acid (24) in humans, and this would lead to a surface expression of the FcR inhibitor (FcγRIIB) on inflammatory cells, thereby attenuating inflammation as it happens with B cells, in which this receptor negatively regulates activating signals (62). It has also been suggested that the effects of IVIg would depend on the activation/polarization state of macrophages; the strongest association between treatments with high doses of IgGs and macrophage polarization seems to depend on Fc receptors of immunoglobulins, which play a major role in macrophage regulation. For our study, it is important to highlight that B lymphocytes from C57BL/6 mice lack this receptor (23) and this could explain the differences in the proportion of LPM and SPM cell subpopulations obtained when mice were challenged with sialylated and non-sialylated ICs when compared to CD1 mice **Figure S12**), in which SPM subpopulation did not increase after the challenges. In this sense, a possible role of this receptor in immune modulation and probably in monocyte migration and transformation into SPM in the peritoneal cavity of C57BL/6 mice is suggested.

Our results reveal an opposite behavior in the expression of IL-1β in both macrophage subpopulations; in this regard, while this cytokine expression was reduced after all the stimuli performed in SPM, in LPM the expression was increased after all of the stimuli employed, especially in ICs formed with sialylated IgGs. However, an increased expression of IFN-γ was only observed in SPM after the challenge with ICs formed with non-sialylated IgGs. Regarding IL-12 and IFN-γ expression in SPM, the levels obtained were under the expression levels of the negative control, except for the IFN-γ expression after the stimulation with non-sialylated ICs which was increased; it is noteworthy that IL-12 induces the expression of IFN-γ (63). On the contrary, LPM stimulated with EVs showed increased levels of IFN-γ when compared to the negative control or when mice were stimulated with sialylated ICs. In the case of IL-12, all the employed stimuli increased the cytokine expression, and for TNF-α, expression levels in all cases were higher than the control in this subpopulation.

IL-18, a cytokine that belongs to the IL-1 superfamily, is produced by macrophages and other cell types (64, 65) and has pleiotrophic roles (66) including the regulation of IL-12 expression (67) and, thus, IFN-γ (68). In monocytes stimulated with IL-12 and IL-18, morphological changes in cells are observed, suggesting the role of these cytokines in differentiation and activation of monocytes into macrophages (69), increasing their phagocytic capacity (70). Under our experimental conditions, a diminished expression of this cytokine was observed in SPM of mice stimulated with EVs and sialylated ICs; however, in LPM, increased expression of this cytokine was observed under the same stimuli. The lower expression levels of IL-12 and the higher expression levels of IFN-γ in SPM of mice stimulated with non-sialylated ICs could correspond to an induction of IL-12 by IL-18, with the consequent induction of IFN-γ as a result of FcγR stimulation by non-sialylated Fc regions of IgGs. Overall results of IL-18 expression suggest that the route of activation of the Th1 response is different in both types of cells and might depend on Fc receptors and on PAMP activation after the phagocytosis of non-sialylated ICs (71).

As IL-18 and IL-12 trigger the production of IFN-γ during an infection, Berclaz et al. evaluated the role of these cytokines in the regulation of FCγR expression in cell membranes by GM-CSF/PU.1, which is related to increased IFN-γ expression in alveolar macrophages (72). In these cells, the expression of IL-18 was augmented by GM-CSF expression *in vivo*, which contributes to the expression of FCγR in the macrophage surface as GM-CSF is able to regulate the constitute expression of this type of receptors and thus, opsonization and phagocytosis. The effect of GM-CSF over FCγ receptors was also studied previously by Rossman et al. (73), revealing a significant increase only in the FCγRII receptor. These studies show how GM-CSF increases FCγRII expression in monocytes, favoring the clearance of the immune complexes; in this sense, a similar mechanism could be employed by LPM of the peritoneal cavity, especially when the stimulation is performed with EVs or sialylated ICs.

Regarding IL-6 expression, increased levels were observed in SPM of EVs-stimulated mice, followed by sialylated ICs; for non-sialylated ICs, undetectable levels were obtained. In the case of LPM of mice challenged with EVs and non-sialylated ICs, higher expression levels were obtained when compared to the control. The role of IL-6 in the immune response toward *T. cruzi* was studied by Sanmarco et al., who compared the expression of this cytokine in wild type mice vs. knockout mice and showed how IL-6 deficient mice produced higher amounts of NO in plasma, accompanied by increases in IL-1β and more inflammatory circulating monocytes (74). In our study, nitric oxide synthase (NOS) expression levels in LPM of mice stimulated with non-sialylated ICs were higher than levels obtained in the other study groups in which IL-6 was not detected, while NOS expression levels in mice stimulated with EVs and sialylated ICs were almost null, while IL-6 expression was increased. IL-6 can potentially exert an non-inflammatory response through inhibition of IL-1 and TNF-α synthesis by macrophages (75, 76), which could be considered a mechanism of regulation of the inflammatory Th17 response at low TGF-β concentrations. Results related to the decrease in the expression levels of cytokines associated to the Th1 response (IL-1, IFN-γ, IL-12 and TNF-α) in SPM, as well as IL-6 expression when mice were stimulated with EVs or sialylated ICs, could be explained by this mechanism.

In this study, the highest expression levels of IL-15 were observed in LPM of mice stimulated with sialylated ICs and in SPM of mice stimulated with EVs of the parasite. This cytokine is a potent autocrine regulator of the production of proinflammatory cytokines by monocytes and macrophages, which, at high concentrations, favors the production of TNF-α, IL-1 and IL-6, while, at low concentrations, favors the production of IL-10 (77). In LPM, the lowest expression of IL-15 induced by EVs and non-sialylated ICs, and the highest expression of IL-10 and TGF-β could be explained by this mechanism. Moreover, the increased expression of IL-15, TNF-α and IL-1β in LPM of mice stimulated with sialylated ICs was not found in SPM. IL-15, in combination with GM-CSF, can induce the differentiation of monocytes into immature dendritic cells (78); this could induce the differentiation of monocytes into SPM. In our study, sialylated ICs recruited more SPM to the peritoneal cavity, while non-sialylated ICs recruited more LPM and larger numbers of dendritic cells were obtained after this stimulus.

IL-10 antagonizes the expression of MHCII, co-stimulatory molecules B7.1/B7.2 (CD80/CD86) and proinflammatory cytokines IL-1β, IL-6, IL-8, TNF-α and, especially, IL-12 (79, 80). SPM is the subpopulation that expresses MHCII to a major extent and, after the challenge of mice with EVs and sialylated ICs, the expression levels of IL-10 presented the highest increase, with decreased expression of IL-1β and IL-12. Moreover, when employing non-sialylated ICs for the challenge of mice, the expression levels of IL-10 as well as other non-inflammatory cytokines is null or under the expression levels of the negative control. In LPM, IL-10 expression after the stimuli with both types of ICs was undetectable, a result that contrasts with the high expression of IL-38 obtained for this type of subpopulation, also considered a regulatory cytokine. In SPM, instead, high levels of IL-10 were observed after the challenge of mice with sialylated ICs; a fact that could be explained as a consequence of the interaction of sialylated groups in ICs with FcRgIIB and thus, the induction of this inhibitory cytokine.

The high expression levels of IL-38 in LPM after the challenge of mice with sialylated ICs contrasts with the absence of expression of this cytokine in SPM. IL-38 is considered an important anti-inflammatory cytokine, which significantly inhibits IL-6, IL-1β, CCL5, TNF-α and CXC10 (related to the Th1 response), as well as IFN-γ (81). From our results, high expression levels of IL-15 and GM-CSF in LPM of mice stimulated with sialylated ICs were observed; however, when the challenge was performed with non-sialylated ICs, this is the only subpopulation in which IL-17 expression levels were higher than in the control, possibly as a consequence of the stimulation through FcγR receptors. The role of IL-17 in Chagas disease has previously been studied and the presence of low parasitemia has been related to the effect of this cytokine (82). In this sense, in infected individuals with the indeterminate form of the disease or with mild cardiopathy, high levels of IL-17 and IL-10 were found, while high levels of IFN-γ and TNF-α were found in patients with severe cardiopathy. In this sense, it seems that IL-17 and IL-10 regulate the Th1 response in order to avoid cardiac involvement (83). LPM of mice challenged with non-sialylated ICs expressed IL-17 but not IL-10.

The results obtained from this work suggest that the immunological tolerance exerted by *T. cruzi* EVs and ICs by macrophages inhibits the response and decreases the secretion of proinflammatory cytokines. Which allow macrophages to control inflammation, avoiding tissue damage caused by an excessive inflammatory response (84) and could be related to the type of immune response that predominates in each phase of the disease (acute or chronic), and thus, to the pathology of chronic Chagas disease, as the Fc region of IgGs that form the ICs interact with FcγR (specially FcγRIIB), eliciting or inhibiting the immune response and favoring (or not) the presence of the parasite and clinic manifestations of the disease. However, the virulence and type of clinic manifestation presented will depend on the parasite strain, as well as the amount of IgGs and the isotypes that could be sialylated as it also happens in other inflammatory or autoimmune diseases.

## Data availability statement

The original contributions presented in the study are included in the article/**Supplementary Material**. Further inquiries can be directed to the corresponding author.

## Ethics statement

The animal study was reviewed and approved by Ethics Committee of the University of Granada (Ethics Committee, 235-CEEA-OH-2018), as well as by the authorities of the Regional Government of the Junta de Andalucía with number 12/11/2017/162.

## Author contributions

Conceptualization, AO, AC-G, and LM; methodology, AC-G, LM, and MG-S; formal analysis, AC-G, LM, and AO; investigation, AC-G, LM, MG-S, and AO, writing—original draft preparation, AC-G, LM, and AO; writing—review and editing, AC-G, AO, LM, and MG-S; visualization, AC-G, AO, and LM; supervision, AO; funding acquisition, AO. All authors contributed to the article and approved the submitted version.
