# Supplementary material for: Extracellular vesicles of Trypanosoma cruzi and immune complexes they form with sialylated and non-sialylated IgGs increase small peritoneal macrophage subpopulation and elicit different cytokines profiles
Source: Front Immunol. 2023 Aug 2;14:1215913. doi: 10.3389/fimmu.2023.1215913 (PMC10434529; doi:10.3389/fimmu.2023.1215913)

Supplementary Material

Extracellular vesicles of *Trypanosoma cruzi* and immune complexes they form with sialylated and non- sialylated IgGs increase small peritoneal macrophage subpopulation and elicit different immunomodulatory responses

Alberto Cornet-Gomez^1 †^, Lissette Retana Moreira ^1, 2, 3 †^, Mercedes Gomez Samblas^1^, Antonio Osuna^1*^

^1^Grupo de Bioquímica y Parasitología Molecular (CTS 183), Departamento de Parasitología, Campus de Fuentenueva, Instituto de Biotecnología, Universidad de Granada, Granada, Spain

^2^Departamento de Parasitología, Facultad de Microbiología, Universidad de Costa Rica, San José, Costa Rica

^3^Centro de Investigación en Enfermedades Tropicales (CIET), Universidad de Costa Rica, San José, Costa Rica

† These authors contributed equally to this work.

*** Correspondence:** Antonio Osuna; aosuna@ugr.es

## Supplementary Figures

**Figure S1**

**
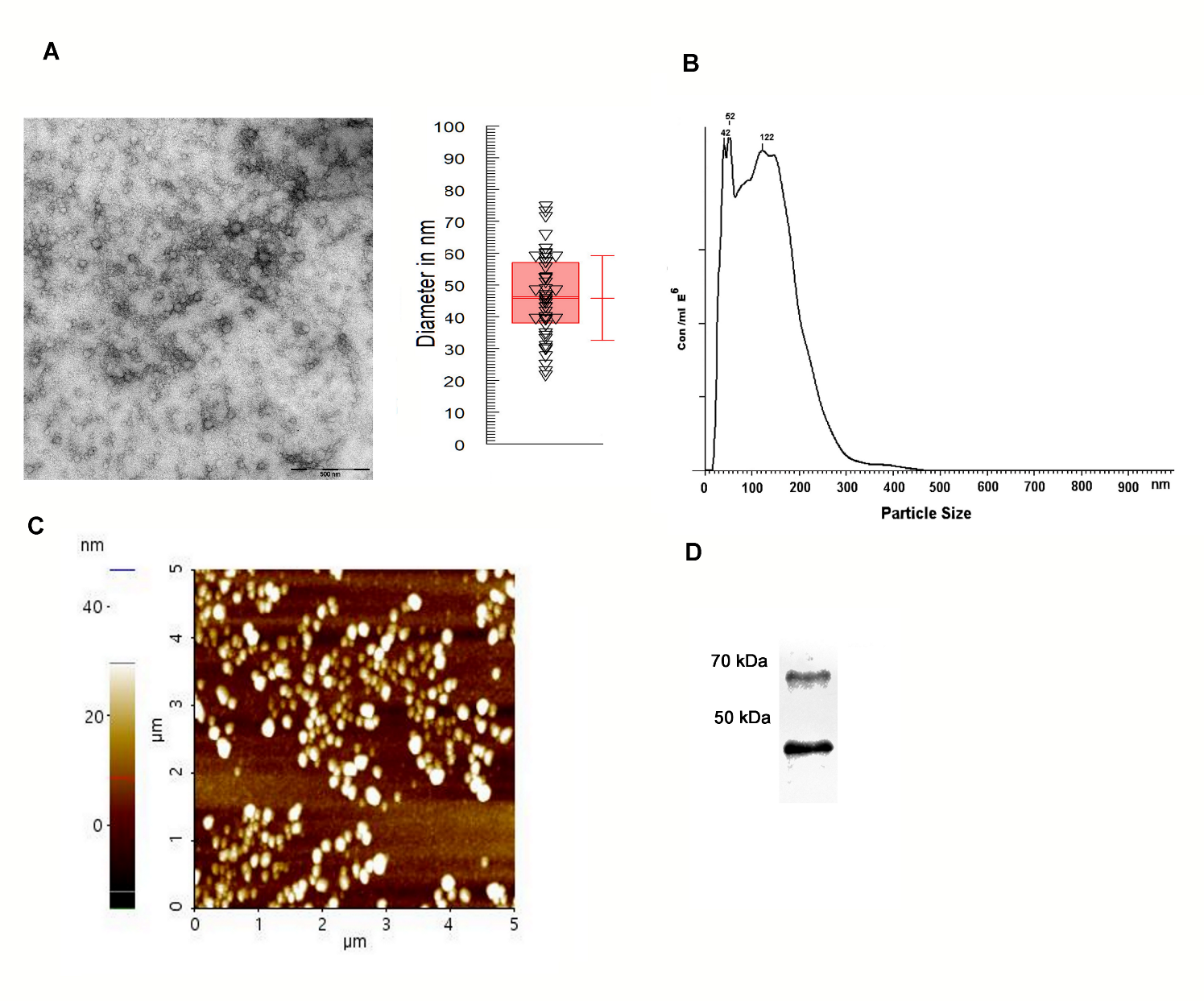
**

**Figure S2. Western blot analysis of cruzipain in extracellular vesicles in trypomastigotes from *T. cruzi***


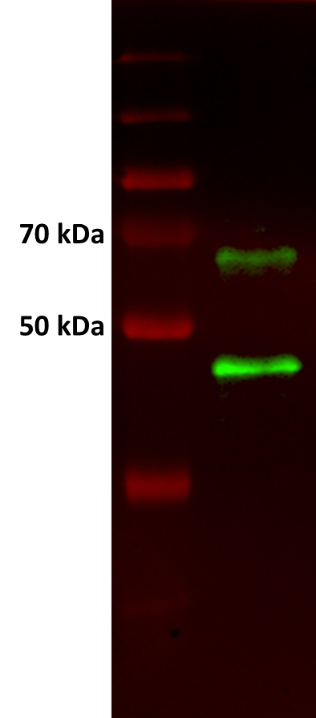


**Figure S3. Western blot analysis of a lysate of trypomastigotes revealed with polyclonal anti-*T.cruzi* antibodies**


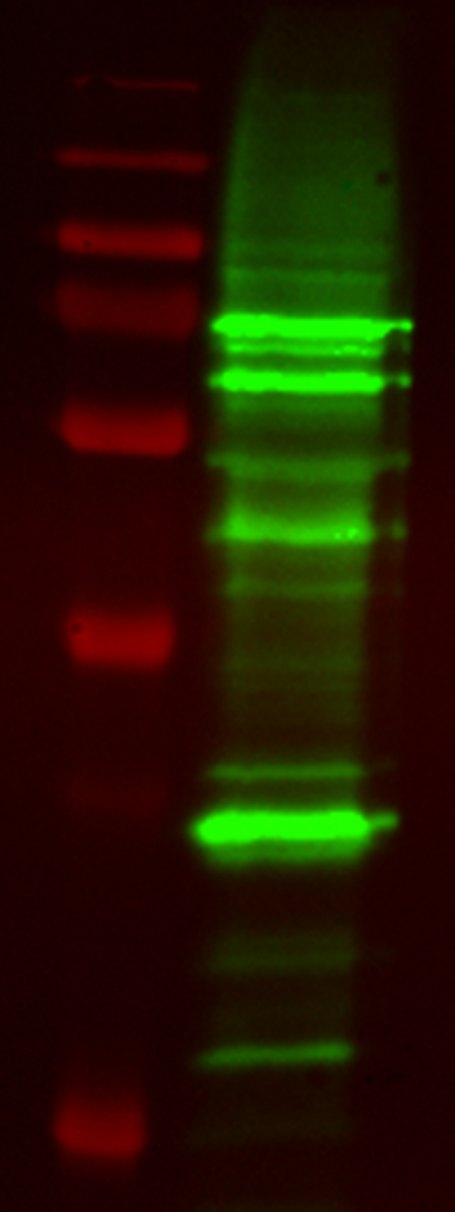


**Figure S4. Immunoglobulin purification**


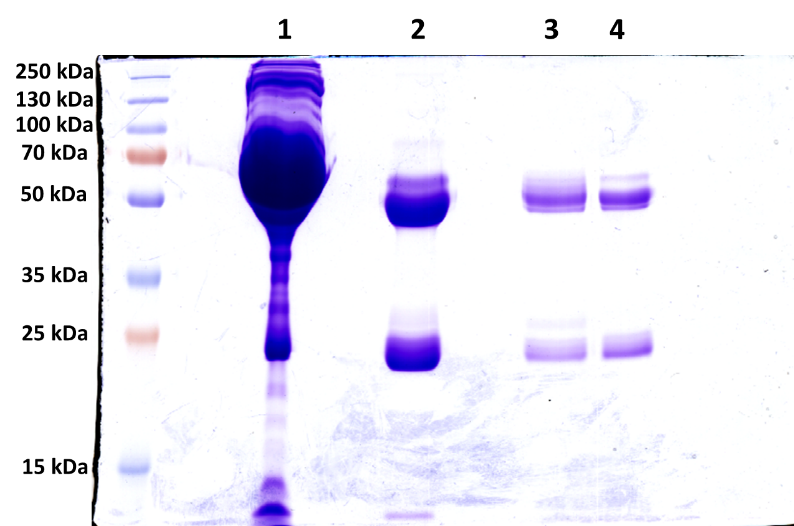


1. **Mouse serum**
2. **IgGs purified with Melon gel**
3. **Sialyzed IgGs**
4. **Non-sialyzed IgGs**

**Figure S5. Western blot analysis of purified IgGs revealed with *Sambucus Nigra* Lectin**

1. **sialylated IgGs**
2. **Non-** **sialylated IgGs**


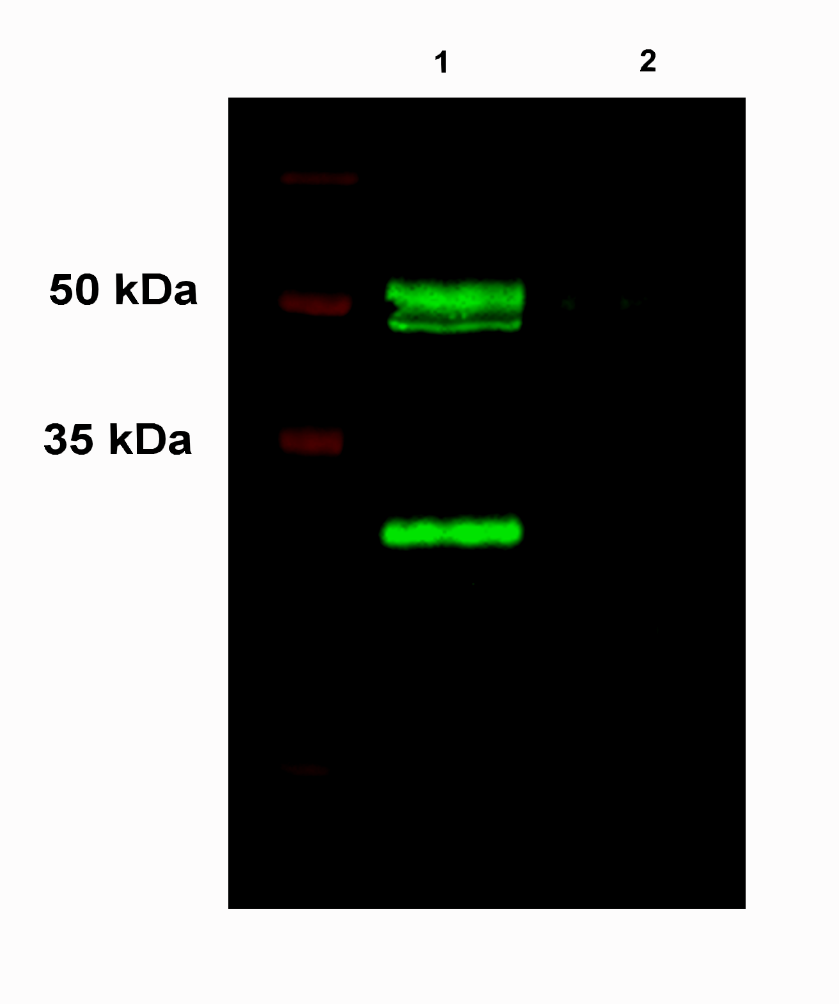


**Figure S6.** Cytograms and gating strategy employed to separate LPM and SPM in PBS-injected mice.

**
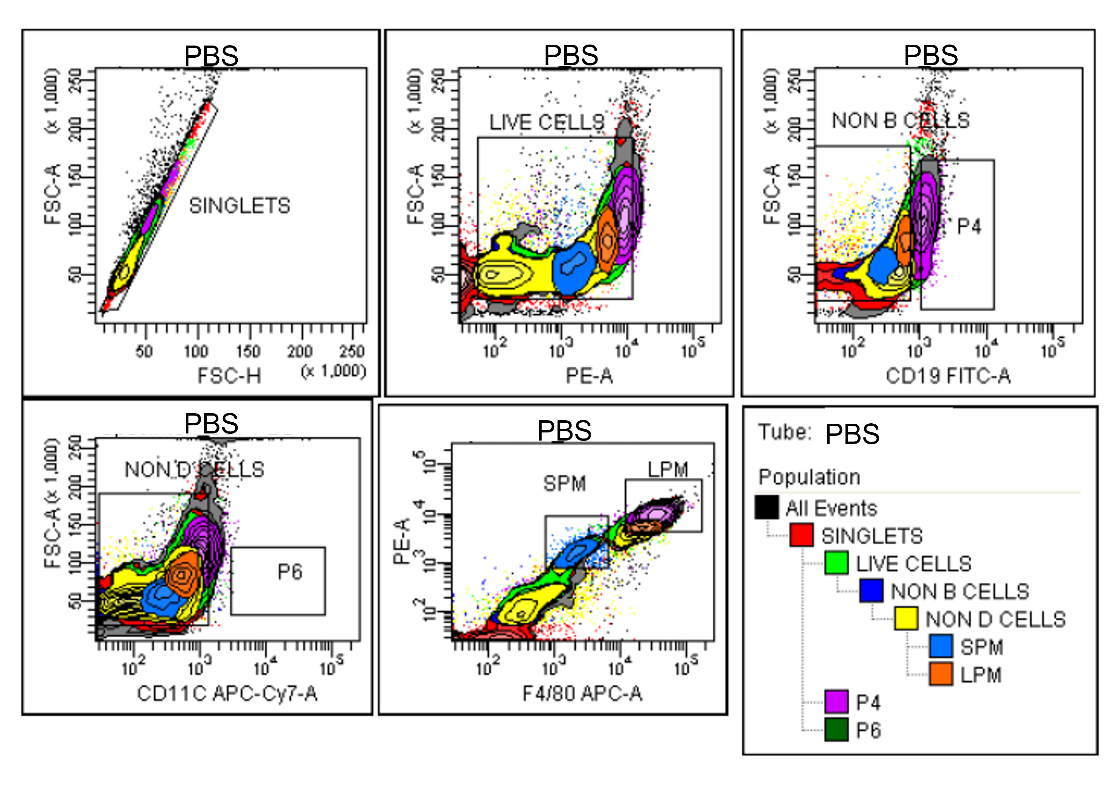
**

**Figure S7.** Cytograms and gating strategy employed to separate LPM and SPM in EVs-injected mice.

**
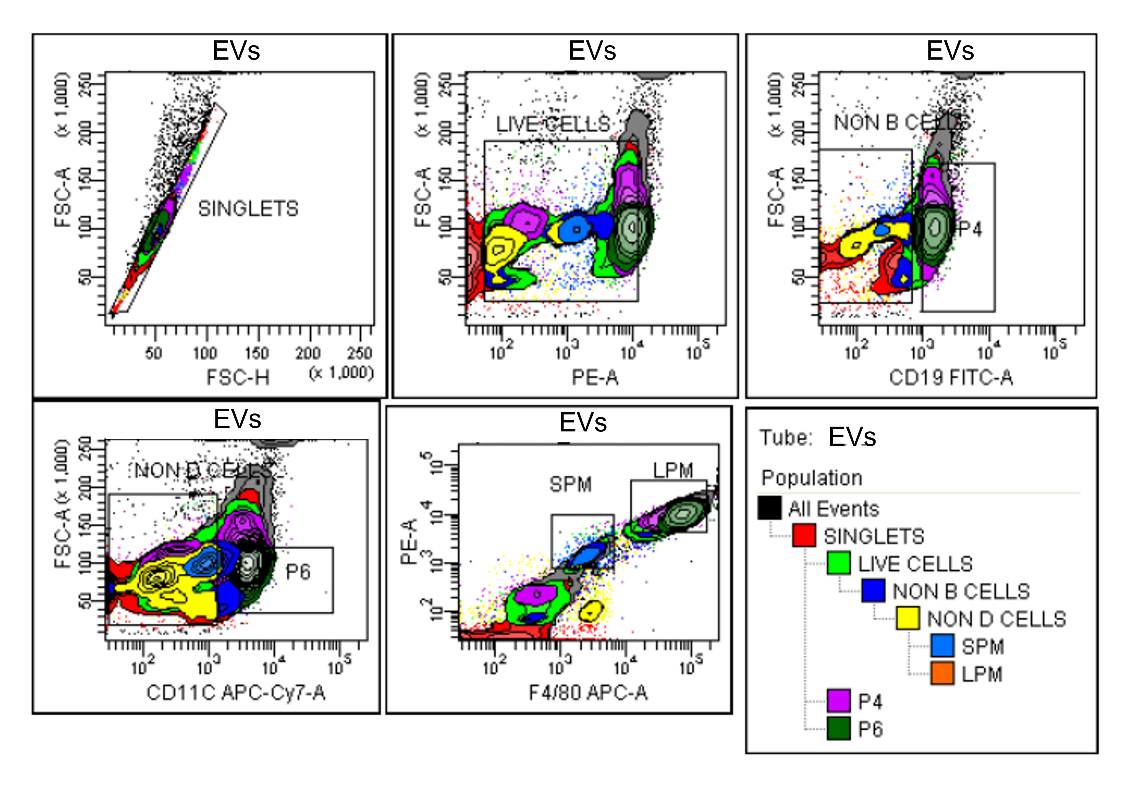
**

**Figure S8.** Cytograms and gating strategy employed to separate LPM and SPM in sialylated ICs-injected mice.

**
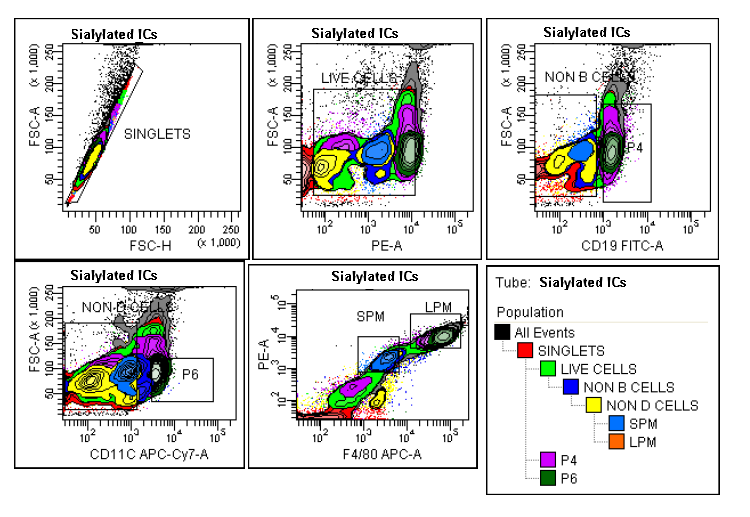
**

**Figure S9.** Cytograms and gating strategy employed to separate LPM and SPM in non- sialylated ICs-injected mice.**
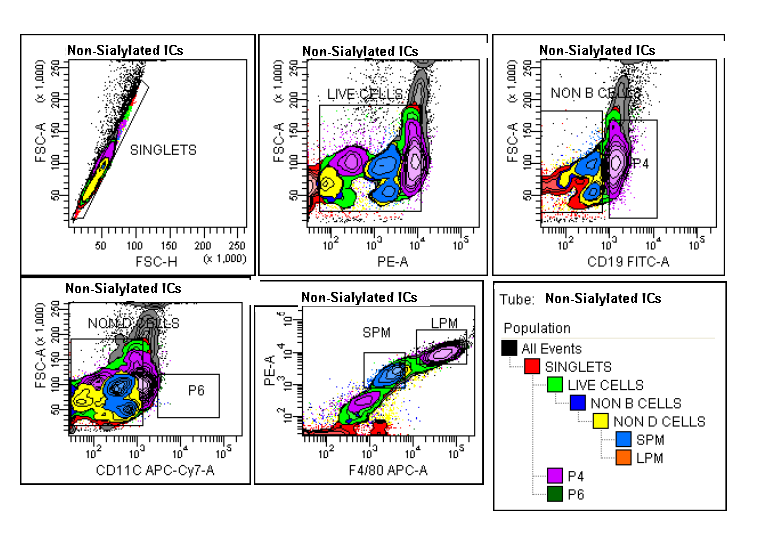
**

**Figure S10.** Cytograms and gating strategy employed to separate LPM and SPM in *T.cruzi*-injected mice.

**
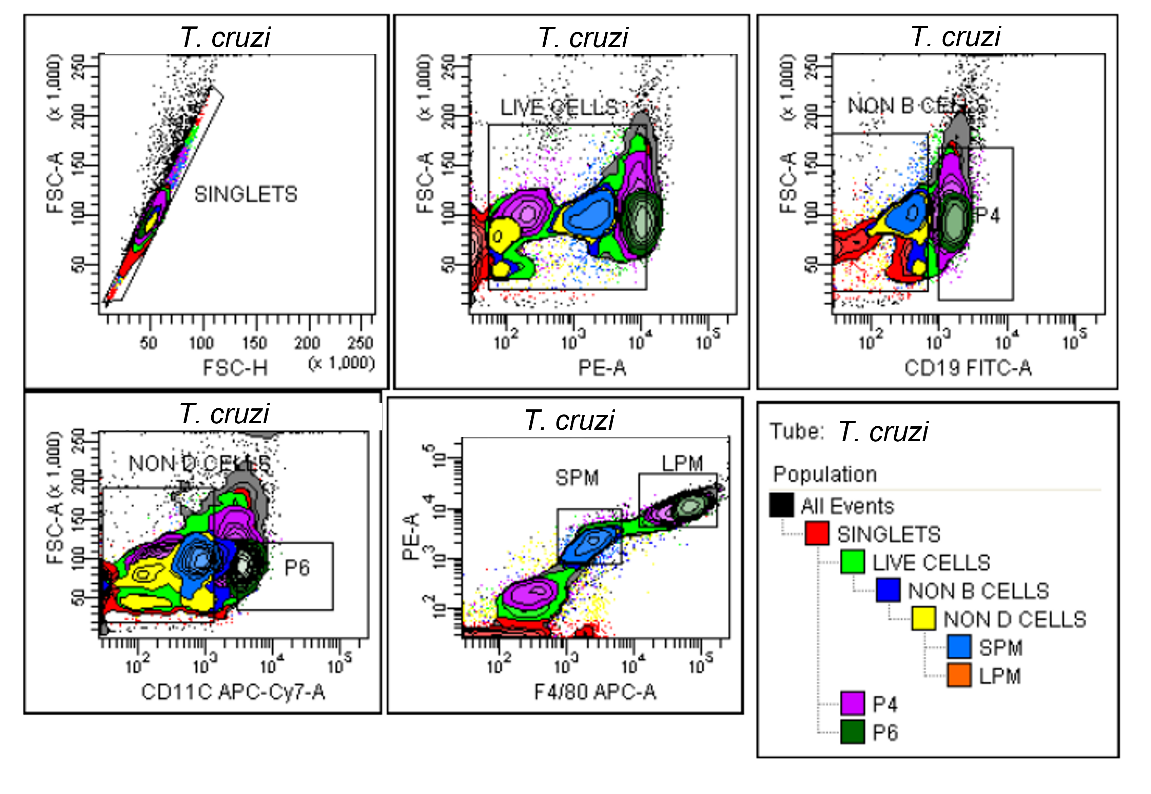
**

**
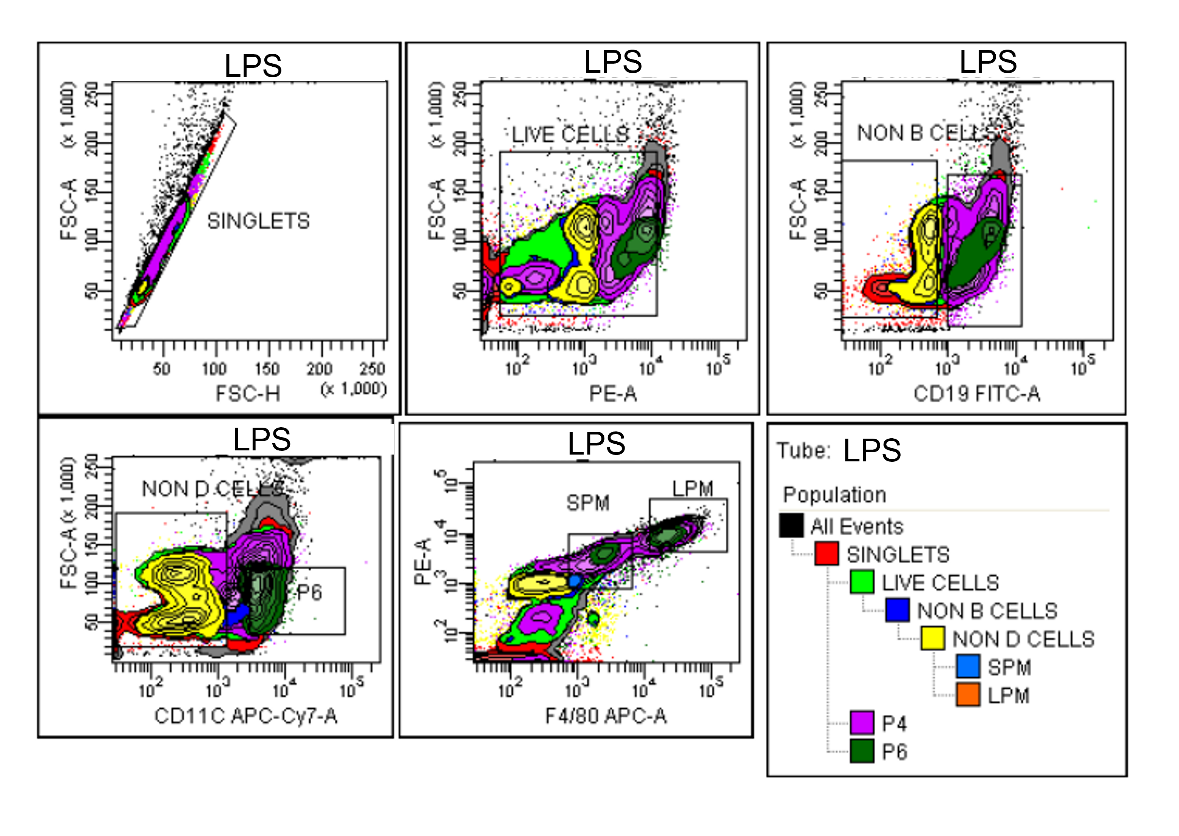
Figure S11.** Cytograms and gating strategy employed to separate LPM and SPM in LPS-injected mice.

**Figure S12.** Percentage of macrophage subpopulations (SPM and LPM) collected from peritoneal cavity of CD1 mice after different stimuli using flow cytometry.





**Figure S13. Heatmap of the relative gene expression of cytokines from Smal Peritoneal macrophages and Large Peritoneal macrophages after the different stimulation**

**
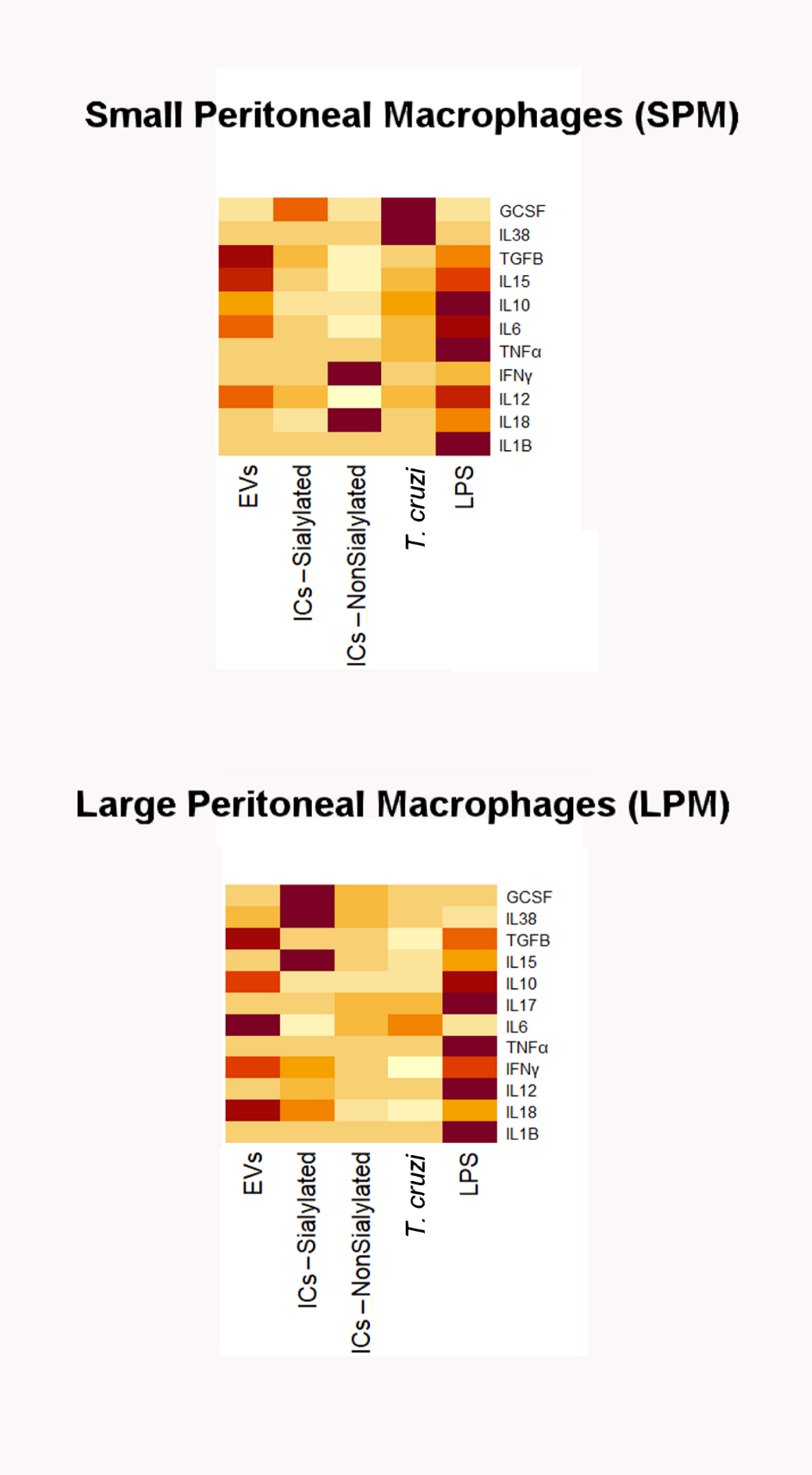
**

## Supplementary tables

| **Antidody** | **Dilution** | **Brand** |
| --- | --- | --- |
| anti-CD19-FITC | 1:50 | Thermo Fischer Scientific, Waltham, MA, USA |
| anti-CD11c-APC-Cy7 clone N418 | 1:50 | Sigma Aldrich, St. Louis, MO, USA |
| anti-rat PE CD11b | 1:20 | BD, Franklin Lakes, NJ, USA |
| F4/80-APC antibody | 1:50 | Miltenyi, Teterow, Germany |
| anti-mouse I-A/I-E (MHCII)- PerCP/Cyanine5.5 | 1:50 | Biolegend, San Diego, CA, USA |

**Table S1**. Antibodies and dilutions employed for the analysis and separation of LPM and SPM, following the protocol previously published by Bou Ghosn et al. (2010)

| **Gene** | **Forward sequence (5'-3')** | **Reverse sequence (5'-3')** |
| --- | --- | --- |
| **IL-1β** | TGCCACCTTTTGACAGTGATG | CTCTTGTTGATGTGCTGCTG |
| **IL-2** | CCACTTCAAGCTCCACTTCA | ATCCTGGGGAGTTTCAGGTT |
| **IL-12** | GACCAAACCAGCACATTGAA | CTACCAAGGCACAGGGTCAT |
| **IL-18** | GACCAAACCAGCACATTGAA | CTACCAAGGCACAGGGTCAT |
| **TNFα** | CCCCAAAGGGATGAGAAGTT | CACTTGGTGGTTTGCTACGA |
| **IFNγ** | CACCCTGAAGTCGTTGTGAA | GATCTCCCCACTCCGGTTAT |
| **G-CSF** | TCCAGGGACTTAAGCAGGAA | CAGAGGCGGATGAAGCTAAT |
| **IL-6** | AGTTGCCTTCTTGGGACTGA | TCCACGATTTCCCAGAGAAC |
| **IL-15** | CATTTTGGGCTGTGTCAGTG | TGCAACTGGGATGAAAGTCA |
| **TGFβ** | TGGAGCAACATGTGGAACTC | AGCCTTGTATCCCGTCTCTT |
| **IL-10** | CAGAGCCACATGCTCCTAGA | TCATTTCCGATAAGGCTTGG |
| **IL-17** | TCCAGAAGGCCCTCAGACTA | TCATGTGGTGGTCCAGCTT |
| **IL-38** | TCAGTATGGGTGGAGGGT | ACAACGGGAGGTGAACAAA |
| **GAPDH** | ATGTGTCCGTCGTGGATC | ACCTGGTCCTCAGTGTAGC |

**Table S2**. Sequences of the forward and reverse primers employed for gene amplification and expression analyses.

|  | **Events** | **Singlets** | **Live cells** | **B Cells** | **Non-B Cells** | **Dendritic Cells** | **Non-dendritic Cells** | **Macrophages** | **SPM** | **LPM** |
| --- | --- | --- | --- | --- | --- | --- | --- | --- | --- | --- |
| **PBS** | 10000 | 5633 | 4924 | 156 | 4768 | 123 | 4645 | 3649 | 506 | 3143 |
| **EV** | 10000 | 8694 | 6394 | 581 | 5813 | 3175 | 2638 | 926 | 444 | 482 |
| **ICs sialylated** | 10000 | 8299 | 5374 | 1915 | 3459 | 343 | 3116 | 1260 | 913 | 348 |
| **ICs non-** **sialylated** | 10000 | 7973 | 5477 | 721 | 4756 | 1986 | 2770 | 2001 | 772 | 1229 |
| ***T.cruzi*** | 10000 | 8761 | 5123 | 368 | 4755 | 799 | 3956 | 1551 | 1028 | 523 |
| **LPS** | 10000 | 8695 | 7116 | 1320 | 5796 | 768 | 5028 | 2038 | 1888 | 150 |

**Table S3**. Results showing the total number of cells belonging to the different populations obtained after each stimulation.

Graphical summary of cytokine expression in large macrophages (LPM) and small macrophages (SPM) after different stimuli.


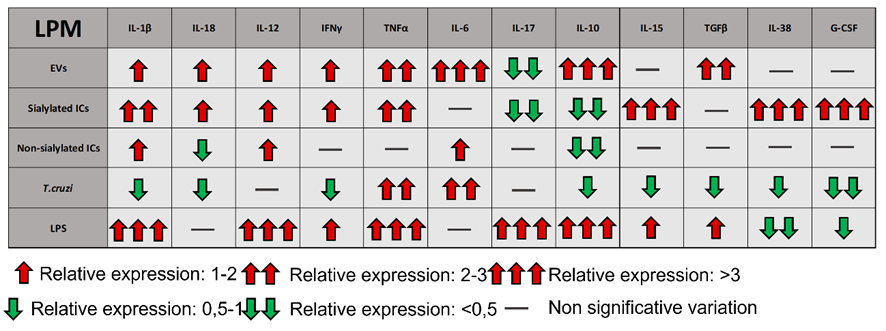


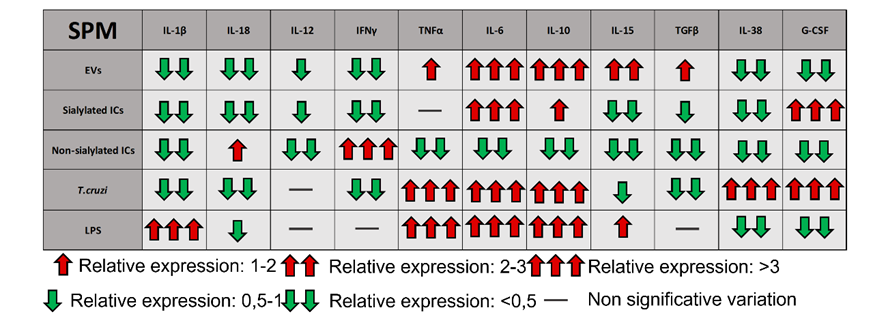

Supplement: Supplementary file 1 [file DataSheet_1.docx]
